# Large scale exploration reveals rare taxa crucially shape microbial assembly in alkaline lake sediments

**DOI:** 10.1038/s41522-024-00537-1

**Published:** 2024-07-28

**Authors:** Zhiguang Qiu, Shuhang He, Chun-Ang Lian, Xuejiao Qiao, Qing Zhang, Ciqin Yao, Rong Mu, Li Wang, Xiao-Ai Cao, Yan Yan, Ke Yu

**Affiliations:** 1 Eco-environment and Resource Efficiency Research Laboratory, School of Environment and Energy, Shenzhen Graduate School, Peking University, Shenzhen, 518055 China; 2 AI for Science (AI4S)-Preferred Program, Peking University, Shenzhen, 518055 China; 3 State Key Laboratory of Isotope Geochemistry, CAS Center for Excellence in Deep Earth Science, Guangzhou Institute of Geochemistry, Chinese Academy of Sciences, Guangzhou, 510640 China

**Keywords:** Microbial ecology, Water microbiology

## Abstract

Alkaline lakes are extreme environments inhabited by diverse microbial extremophiles. However, large-scale distribution patterns, environmental adaptations, community assembly, and evolutionary dynamics of microbial communities remain largely underexplored. This study investigated the characteristics of microbial communities on rare and abundant taxa in alkaline lake sediments in west and northwest China. We observed that abundant taxa varied significantly with geographical distance, while rare taxa remained unaffected by regional differences. The assembly process of abundant taxa was influenced by dispersal limitation, whilst rare taxa were predominantly driven by heterogeneous selection. Network analysis indicated that rare taxa as core species for community interactions and community stability. Rare taxa exhibited higher speciation and transition rate than abundant taxa, serving as a genetic reservoir and potential candidates to become abundance taxa, highlighting their crucial role in maintaining microbial diversity. These insights underscore the significant influence of rare taxa on ecosystem biodiversity and stability in alkaline lakes.

## Introduction

Microorganisms are key components in lake ecosystems that are crucially involved in the ecosystem functions^1,2^. Lake sediments are rich in organic and inorganic matters that directly or indirectly shape microbial communities^3,4^. Specifically, sediment microbial communities in alkaline lakes have adapted to thrive in extreme environmental conditions, such as high pH and often high salinity. These extremophiles interact intricately with each other with specialised metabolic activities that are crucial to the biogeochemical cycles of alkaline lakes^5^. This adaptation to extreme environments has led to unique microbial biodiversity hotspots, which are critical in maintaining the health and functionality of these ecosystems, as well as their biogeochemical cycles^6^. Therefore, investigating microbial communities in alkaline lake sediments enables valuable insights into the resilience and adaptability of microbial life in extreme environments.

In certain microbial communities, distinct niches are occupied by different microorganisms, characterised by their taxonomy and interactions with other organisms, leading to different abundances and various ecological functions within specific habitats. Exploring the differences of ecological characteristics and strategies between microbial taxa in terms of different abundances, namely abundant and rare taxa along with species co-occurrences can assist decoding the microbial assemblages, microbial functions, and dynamics of community succession in certain environments^7,8^. For example, previous studies reported that abundant taxa in the community focus on the microbial role of biogeochemical cycles such as photoautotrophic carbon fixation^9,10^ and the decomposition of soil organic matters^11^. In contrast, rare taxa are critical in facilitating ecosystem functions such as improving functionalities of the communities and enhancing resilience to environmental disturbances^12^. Such analyses on abundant and rare taxa have been applied in various environments such as soil, freshwater, marine, and hot springs^13–16^. However, there is still a lack of comprehensive studies of both abundant and rare taxa within the alkaline lake ecosystems on a large geographic scale.

Although abundant and rare taxa co-occur in a given habitat, many studies have shown that these two subcommunities often exhibit distinct ecological traits in terms of distribution, assembly, and co-occurrence patterns^17–19^. The assembly of abundant and rare taxa is shaped by environmental factors, which is known as deterministic processes. For example, one previous study suggested that pH and average annual temperature mainly drive the assembly of abundant and rare taxa in soil environments, respectively^20^. However, the assembly of these taxa can also be dominated by diffusion constraints, namely stochastic processes^21,22^. These analyses of microbial communities in response to environmental factors is crucial to predict their biogeographical distribution, evolutionary adaptabilities, and community dynamics under climate changes. Additionally, analyses of microbial interactions based on co-occurrence networks also provide insights into abundant or rare taxa as keystone taxa in microbial networks, promoting the maintenance of community and functional stability^23^. While some studies emphasised that abundant taxa may be crucial in the assembly process, recent studies suggested that rare taxa are also vital in the maintenance of ecological stability^20,24–26^. Several studies investigated many lakes in Qinghai-Tibet Plateau or Inner Mongolia-Mongolian Plateau^27,28^, have significantly enhanced our understanding of geological, ecological, and microbial dynamics within these alkaline lakes. However, research in large-scale exploration of alkaline lake microbiota, and their ecological characteristics and ecological value of abundant and rare taxa across the expansive western-northern region of China, is still limited.

To decode the assembly mechanisms and predict the dynamics of alkaline sediment microbial community, we investigated 32 alkaline lakes in Qinghai-Tibet Plateau, Inner Mongolia, and Xinjiang Uygur Autonomous Region in China, with pH ranged from 7.1 to 11.3. We explored alkaline lake sediment microbial communities in terms of abundant and rare taxa and analysed microbial characteristics of environmental adaptation, community aggregation, potential interactions, and evolutionary traits between abundant and rare taxa, aiming to uncover the role of abundant and rare taxa in the community formation and maintenance of ecosystem diversity and stability in alkaline lake sediment, providing valuable knowledge for biodiversity conservation and ecosystem management.

## Results

### Distribution patterns of abundant, intermediate, and rare taxa

After removal of amplicon sequence variants (ASVs) < 25 reads, a total of 8,899,202 purified reads were classified into 25,885 ASVs across all samples, including 111 ASVs identified as regionally abundant taxa (relative abundance > 0.1% across all samples), 10,661 ASVs as intermediate taxa (relative abundance between 0.001% and 0.1%), and 15,113 ASVs as rare taxa (relative abundance ≤ 0.001%). Generally, an average of 58.4% ASVs were considered as rare taxa, but these ASVs only accounted for 8.4% of the average relative abundance across all samples. Conversely, only an average of 0.4% ASVs were considered as abundant taxa, but these ASVs accounted for approximately 30.0% of the relative abundance across all samples. Intermediate taxa had similar proportion of both relative abundance and richness (Supplementary Fig. 2a). Among these ASVs, the top abundant phyla in abundant taxa were *Firmicutes* (40.6%), *Bacteroidetes* (11.4%), and *Actinobacteria* (10.7%). Intermediate taxa had similar microbial structure at phylum level to abundant taxa, but the top abundant phyla in rare taxa were *Proteobacteria* (28.0% of the whole rare sub-community), *Firmicutes* (13.5%), and *Bacteroidetes* (12.0%) (Supplementary Fig. 2b), suggesting that abundant and rare taxa had distinct patterns of community structure in alkaline lake sediments. Functional prediction results showed that abundant taxa and rare taxa contributed similarly to carbon fixation pathways in terms of the percentage of their total genes, respectively, while such predicted genes are more abundant in abundant taxa than in rare taxa, possibly linked to their relative abundances of the overall communities (Supplementary Table 5).

**Fig. 1:**
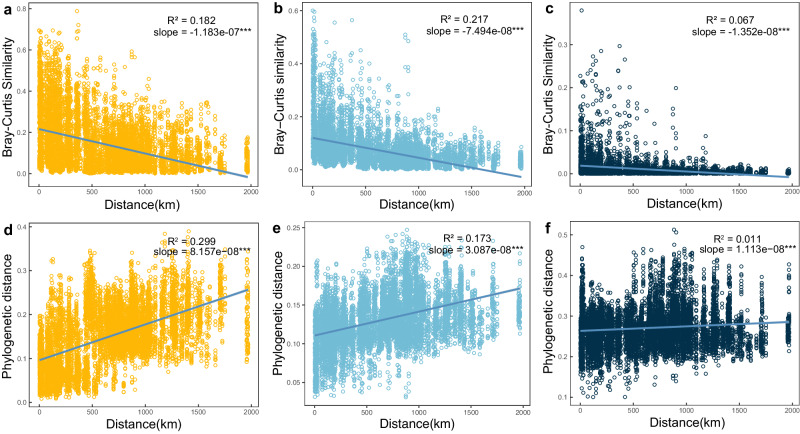
Biogeographical patterns and phylogenetic diversity of abundant, intermediate, and rare taxa in alkaline lake sediments. Distance-decay curves of abundant taxa (**a**), intermediate taxa (**b**), and rare taxa (**c**) based on Bray-Curtis similarity. Distance-decay curves of abundant taxa (**d**), intermediate taxa (**e**), and rare taxa (**f**) based on phylogenetic distance. Significant differences with *P*-values determined using linear regression model, marked by *** for *P* < 0.001, and cyan lines indicate best linear fit.

The estimation of biogeographical and phylogenetic patterns in sediment samples showed that the Bray-Curtis similarity distances of abundant, intermediate, and rare communities were negatively correlated with geographic distances (*P* < 0.001), in which the fitness values of abundant taxa were higher than intermediate and rare taxa (R^2^ < 0.1). The slope of abundant taxa was steeper than that of rare taxa (Fig. 1a, c), suggesting that abundant taxa had a strong decay of the community similarity with geographic distance in sediment samples, and abundant taxa have higher spatial species turnover rate than the rare ones. As for phylogenetic distances based on beta mean nearest taxon distance (β-MNTD) matrices, both abundant and rare taxa showed significantly (*P* < 0.001) positive correlations, similar to the trend of the distance decay relationship (DDRs), but abundant taxa had higher fitness values and steeper slope than rare taxa (Fig. 1d, f), suggesting that the phylogenetic structure of abundant taxa had greater variation with the increase of geographical distance compared to rare taxa. Intermediate taxa in Bray-Curtis similarity and phylogenetic distances were found as intermediate trends compared to abundant and rare taxa (Fig. 1b, e).

### Environmental responses of abundant, intermediate, and rare taxa

**Fig. 2:**
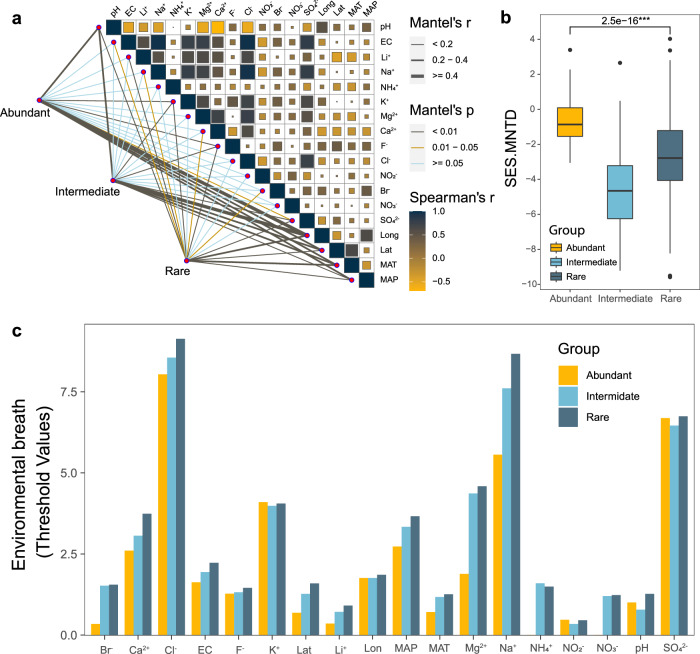
Effects of environmental factors on microbial composition of abundant, intermediate, and rare taxa and their environmental adaptability in alkaline lake sediments. **a** Spearman’s correlation of environmental factors with abundant (yellow), intermediate (cyan), and rare taxa (navy) based on Mantel tests. **b** the difference in SES.MNTD value among abundant, intermediate, and rare taxa. Significant differences with P-values were determined using Wilcoxon rank sum test, marked by *** for *P* < 0.001. The boxplot shows the distribution of data, the central dot in the box represents the median, the box bounds represent the 25th and 75th percentiles, and whiskers represent the minima to maxima values. **c** Environmental adaptation of abundant (yellow), intermediate (cyan), and rare taxa (navy) in sediments of alkaline lakes. Environmental breadths were assessed by the threshold values of abundant, intermediate, and rare taxa in response to environmental factors based on TITAN, respectively. Lon Longitude, MAP Mean annual precipitation, MAT Mean annual temperature. Units of parameters: mg/L (Br^−^, Ca^2+^, Cl^−^, F^−^, K^+^, Li^+^, Mg^2+^, Na^+^, SO_4_^2−^, NO_2_^−^, NO_3_^−^, and NH_4_^+^), ms/cm (EC), °C (MAT), and mm (MAP).

To unravel the correlations of environmental factors with biogeographical and phylogenetic patterns of the microbial communities, we further investigated the responses of abundant, intermediate, and rare taxa to environmental changes. A Mantel test between community composition and environmental factors showed that the community composition of abundant, intermediate, and rare taxa was all significantly correlated with physicochemical factors such as pH, K^+^, F^+^, and SO_4_^2-^, as well as geoclimatic factors such as longitude, latitude, mean annual temperature (MAT), and mean annual precipitation (MAP) (*P* < 0.05) (Fig. 2a). In addition, the community composition of rare taxa was also significantly correlated with Li^+^, Ca^2+^, Cl^-^, and Br^-^ (*P* < 0.05), but no significant correlation was found in abundant taxa on these parameters (Fig. 2a).

To assess the effects of environment filtering on communities, standardized effect size of mean nearest taxon distance (SES.MNTD) was calculated. Results showed that the mean values of SES.MNTD in rare taxa (mean = −2.725) were significantly lower than abundant taxa (mean = −0.639) (Wilcoxon, *P* < 0.001), while intermediate taxa were significantly lower than the other two subcommunities (mean = −4.636) (Wilcoxon, *P* < 0.001) (Fig. 2b), suggesting that the phylogenetic structure of rare taxa and intermediate taxa were more clustered than that of abundant taxa. Further estimation on the environmental thresholds using Threshold Indicator Taxa Analysis 2 (TITAN2) showed that rare taxa had a broader range of environmental thresholds than abundant taxa in most of the environmental factors except K^+^ and NO_2_^-^, while environmental thresholds of intermediate taxa were found to be in the middle between abundant and rare taxa across all environmental factors except K^+^, NO_2_^-^ pH, and SO_4_^2-^ (Fig. 2c, Supplementary Figs. 3, 4), suggesting that rare taxa may have stronger adaptability to the alkaline lake environment. The above results suggested that the community composition of abundant, intermediate taxa, and rare taxa was influenced by different environmental variables, while the rare taxa showed broader environmental adaptation ability, and were more likely to be influenced by environmental filtering than abundant taxa.

### Assembly processes in abundant, intermediate, and rare taxa

**Fig. 3:**
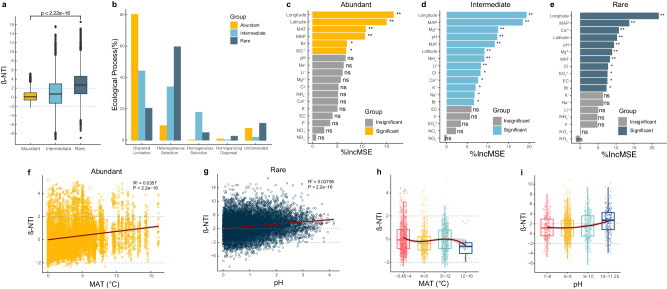
Ecological processes shape the community assembly of abundant, intermediate, and rare taxa in alkaline lake sediments. **a** Null model analysis of deterministic and stochastic processes on the assemblies of abundant (yellow), intermediate (cyan), and rare taxa (navy) in alkaline lake sediments. Significant differences with *P*-values were determined using Wilcoxon rank sum test, marked by *** for *P* < 0.001. The boxplot shows the distribution of data, the central dot in the box represents the median, the box bounds represent the 25th and 75th percentiles, and whiskers represent the minima to maxima values. **b** iCAMP analysis of five ecological processes on the assemblies of abundant (yellow), intermediate (cyan), and rare taxa (navy). The five ecological processes including stochastic processes (homogenizing dispersal, dispersal limitation, and undominated part) and deterministic processes (heterogeneous selection and homogenizing dispersal). **c**–**e** Environmental factors correlated to abundant (**c**), intermediate (**d**), and rare (**e**) taxa listed in descending order of importance to community assembly based on Random Forest analysis. Significance of *P*-values were marked by * for *P* < 0.05, ** for *P* < 0.01. **f**, **g** The relationship between β-NTI and differences in MAT for abundant taxa (**f**) and pH for rare taxa (**g**). Linear regressions models in red lines with associated correlation coefficients were given on each panel. Horizontal dashed lines indicate the β-NTI significance thresholds of +2 and −2. **h**, **i** Patterns of β-NTI across different categories in MAT for abundant taxa (**h**) and pH for rare taxa (**i**) The boxplot shows the distribution of data, the central dot in the box represents the median, the box bounds represent the 25th and 75th percentiles, and whiskers represent the minima to maxima values. Red lines indicate the cubic linear fit, and grey areas indicate 95% confidence intervals.

To explore the differences in assembly strategies of microbial communities in response to different environmental factors, we estimated the contributions of stochastic and deterministic processes to community assembly of abundant, intermediate, and rare taxa based on null model analysis. Results showed that community assembly of abundant taxa was dominated by stochastic process (90.0%), while rare taxa was mainly governed by deterministic assembly (64.8%). Contributions of stochastic process (52.7%) and deterministic process (47.3%) were similar in community assembly of intermediate taxa (Fig. 3a). Infer Community Assembly Mechanisms (iCAMP) analysis showed that dispersal limitation belonging to stochastic process dominantly contributed to abundant taxa assembly (80.64%), followed by heterogeneous selection (9.63%), homogeneous selection (0.52%), homogenizing dispersal (1.24%), and undominated part (7.96%) (Fig. 3b). Heterogeneous selection in the deterministic process was mainly found in rare taxa assembly (59.99%), followed by dispersal limitation (20.78%), homogeneous selection (5.09%), homogenizing dispersal (2.91%), and undominated part (11.23%) (Fig. 3b). In intermediate taxa, dispersal limitation was the main contributor (44.61%), followed by heterogeneous selection (34.42%), homogeneous selection (18.03%), homogenizing dispersal (0.63%), and undominated part (2.31%) (Fig. 3b). These results indicated that the community assembly process of abundant taxa was mainly influenced by stochastic processes, such as dispersal limitation which linked to geographical distance, while the assembly of rare taxa was mainly influenced by deterministic processes, such as environmental variable selection. The assembly of intermediate taxa was not dominated by either stochastic process or deterministic process.

To investigate the effects of environmental factors on the community assembly process, a random forest (RF) analysis based on beta Nearest Taxon Index (β-NTI) dimension reduction data and environmental data was conducted. Results showed that longitude, latitude, MAT, MAP, Br^-^, and SO_4_^2-^ significantly influenced abundant taxa (*P* < 0.05), while pH, Ca^2+^, Mg^2+^, Cl^-^, and EC significantly influenced rare taxa, in addition to the factors that significantly influenced abundant taxa (*P* < 0.05). Markedly, intermediate taxa were influenced by nearly most of the environmental factors except EC, F^-^, SO_4_^2-^, NO_2_^-^, and NO_3_^-^, suggesting that based on the environmental variables measured, rare and intermediate taxa were more sensitive to environmental factors than that of abundant taxa (Fig. 3c–e). To further evaluate the influence of each environmental factor on deterministic and stochastic community assembly processes, environmental variables, and geospatial factors against β-NTI of abundant and rare taxa were estimated using Mantel test. Results showed that the assembly process of abundant taxa was mainly correlated to MAT, pH, Na^+^, and spatial (*P* < 0.001), while the assembly process of rare taxa was mainly correlated to pH, EC, Na^+^, K^+^, F^-^, Cl^-^, MAT, MAP, and spatial (*P* < 0.001). The assembly process of intermediate taxa was significantly correlated to almost factors except NH_4_^+^, NO_2_^-^, and NO_3_^-^ (Supplementary Table 2). Partial mantel test to confirm the optimal predicted environmental factors showed that after controlling other environmental factors, pH was the best environmental predictor of rare taxa (Supplementary Table 3), while longitude, latitude, and MAT were environmental predictors of abundant taxa (Supplementary Table 4). Notably, no best environmental factor can be chosen to predict intermediate taxa after controlling other environmental factors (Supplementary Table 6), therefore further analysis was only based on abundant and rare taxa. Analysis of the correlation between β-NTI and environmental factors showed that the relative contribution of deterministic processes increased along with the increase of pH in rare taxa, while the relative contribution of deterministic processes tended to increase when MAT is above 12 °C in abundant taxa (Fig. 3e). In terms of the correlation between β-NTI and the changes of sediment MAT and pH (i.e., delta value of MAT and pH), β-NTI of abundant and rare taxa were both significantly (*P* < 0.01) and positively correlated with the delta value of sediment MAT and pH, respectively (Fig. 3d), suggesting that the community assembly process of abundant taxa tended to shift from stochastic to deterministic process with the increase of the changes in MAT, while rare taxa tended to shift from stochastic to deterministic process with the increase of the changes in pH.

### Co-occurrence patterns of abundant, intermediate, and rare taxa

**Fig. 4:**
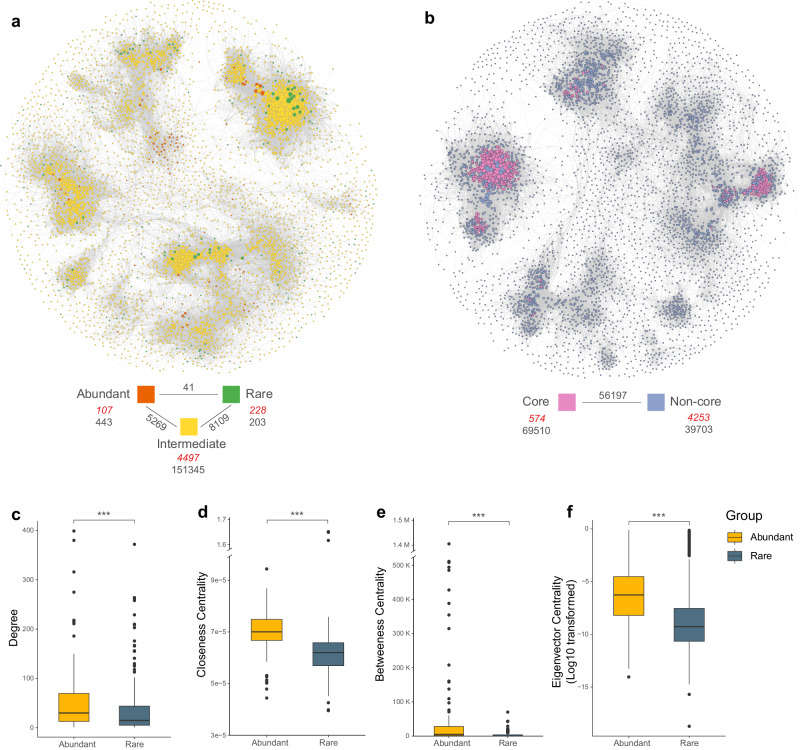
Co-occurrence network analysis of the microbial communities in alkaline lake sediments. **a** The coexistence patterns among abundant (orange), rare (green), and intermediate taxa (yellow). **b** The coexistence patterns between core (pink) and non-core (blue) microbial communities. The size of each node represents proportional to the node degree of the ASVs. The black numbers below each taxa group indicate the number of connections within the group, and the black numbers between coloured blocks indicate connections between groups. Red italicised numbers indicate the number of nodes in each group. Comparison of node-level topological characteristics of abundant (yellow) and rare taxa (navy), including degrees (**c**), closeness centrality (**d**), betweenness centrality (**e**), and eigenvector centrality (Log10 transformed, **f**). The boxplot shows the distribution of data, the central dot in the box represents the median, the box bounds represent the 25th and 75th percentiles, and whiskers represent the minima to maxima values.

To explore the co-occurrence pattern of the sediment microbial community, a meta-community co-occurrence network was established. The network analysis captured 165,410 edges among 4,827 nodes, in which 107 and 228 nodes were attributed to abundant and rare taxa, respectively. There were 203 inner associations found within rare taxa, 443 within abundant taxa, and 151,345 within intermediate taxa (Fig. 4a). Only 41 inner associations were found between abundant and rare taxa, but 8109 connections were observed between rare and intermediate taxa, and 5269 connections were found between abundant and intermediate taxa, suggesting that rare taxa were more frequently co-occurred with intermediate taxa than co-occurred with abundant taxa. In the context of core communities (nodes with degree > 100 and betweenness centrality values < 5000) and non-core communities, a total of 69,510 and 39,703 inner associations were found in core communities and non-core communities, respectively, and 56,197 connections were found between the two groups (Fig. 4b). In this network, a total of 574 ASVs including 15 rare taxa and 5 abundant taxa were classified as network core species (nodes with degree > 100 and betweenness centrality values < 5000). In abundant network core species, two ASVs were affiliated to phylum *Halanaerobiaeota*, and others belonged to phyla *Acetothermia*, *Chloroflexi*, and *Actinobacteria*. On the other hand, 15 rare network core species were affiliated with phyla *Proteobacteria*, *Firmicutes*, *Bacteroidetes*, *Actinobacteria*, *Cyanobacteria*. Rare taxa were more likely to be found as network core species, which were approximately three times as many as abundant taxa.

To further evaluate the role of abundant and rare taxa in maintaining the network stability, we determined the values of topological parameters of abundant and rare taxa, including degree, closeness, betweenness, and eigenvector centrality in the network analysis. These values of abundant taxa were found significantly higher than rare taxa (Fig. 4c–f), suggesting that abundant taxa were often found in central positions within the network, which were likely to be functioned in maintaining the ecosystem stability, while rare taxa were more frequently found as network core species which crucially involved in species interactions. The values of topological parameters for intermediate taxa were found in the middle between abundant and rare taxa (Supplementary Fig. 5), suggesting that intermediate taxa may play an intermediate role in the network topology.

### Evolutionary shifts between abundant, intermediate, and rare taxa

**Fig. 5:**
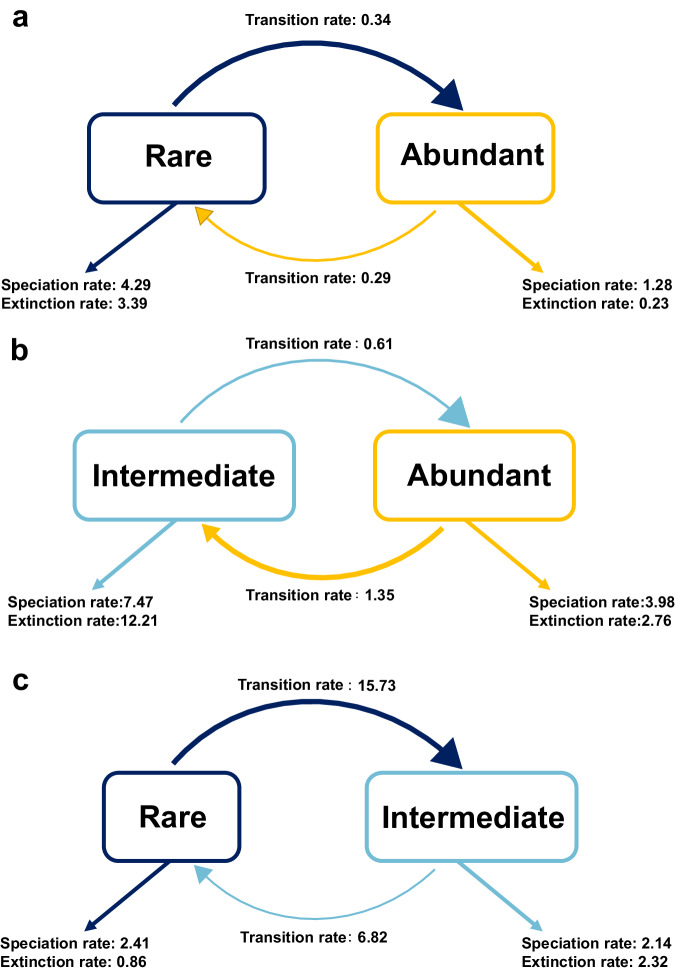
Graphical representation of the BiSSE model. Estimation of evolutionary characteristics between **a** abundant and rare taxa, **b** abundant and intermediate taxa, and **c** rare and intermediate taxa.

To predict the community dynamics in the future scenarios, we defined abundant, intermediate, and rare as different ecological states in the Binary-state speciation and extinction (BiSSE) model to determine the speciation rate and extinction rate of abundant, intermediate, and rare taxa, respectively, as well as the transition rate between the two ecological states. Result showed that rare taxa (speciation rate: 4.291, extinction rate: 3.3386) were predicted as about 3-fold higher speciation rate and 15-fold higher extinction rate than abundant taxa (speciation rate: 1.437, extinction rate: 0.233), while the transition rate from rare taxa to abundant taxa (0.338) was higher than that from abundant taxa to rare taxa (0.287), suggesting that rare taxa had a higher probability of transitioning to abundant taxa than the conversed direction (Fig. 5a). The transition rate from abundant to intermediate taxa (1.35) was higher than that from intermediate to abundant (0.61), while the transition rate from rare to intermediate taxa (15.73) was higher than the opposite direction (6.82) (Fig. 5b, c), further suggesting that rare taxa could be a potential candidate as taxonomic repository in the environment. Overall, rare taxa could be potential candidates of abundant taxa, where rare taxa have the potential to become abundant taxa under radical environmental disturbances to maintain community stability and improve the adaptability of the microbial community.

## Discussion

The exploration of microbial communities in terms of their biogeographic distribution, environmental adaptation, community assembly processes, and evolutionary characteristics can improve our understanding on the functions and ecological roles of microorganisms^29–32^. Specifically, studying microbial communities in the context of their specific niches can help understand microbial interactions and succession under diverse environmental conditions^9,33,34^. In this study, we surveyed microbial communities from 136 alkaline lake sediments, across Qinghai-Tibet Plateau, Inner Mongolia, and Xinjiang Uygur Autonomous Region, covered more than 2/3 of the saline lake region in China (Supplementary Fig. 1). Although sampling of alkaline lake sediments was conducted at 0–10 cm depth, which potentially generate mixed microbial communities across the range of sampling depth and neglect the physiochemical gradients at the centimetre scale^35,36^, this study focused on general microbial assemblages of alkaline lake sediments under a large scale, following similar approaches on lake sediment studies^37–41^. We employed a range of analytical techniques, including DDR analysis, community assembly analysis, TITAN, co-occurrence network analysis, and the BiSSE model, to investigate the three distinct groups of microbial communities: the abundant, intermediate, and rare taxa in alkaline lake sediments. These analyses decoded the patterns of geographical distribution and the characteristics of microbial communities that respond to environmental changes on a large scale. Specifically, we found that the microbial assemblage strategies of abundant, intermediate, and rare taxa were influenced by different environmental factors, consequently formed distinct geographical patterns across the saline lake region of northwestern China.

While several studies focused on generalists and specialists in microbial communities^30,42,43^, our study investigated abundant, intermediate, and rare taxa following the methods used in a hot spring study^44^ to dissect the ecological roles of microbial communities under different niches. We used a cutoff at < 0.001% of the relative abundance for rare taxa, following a majority of the previous studies^17,45–48^. As abundant taxa may vary distinctly across geographical distances, previous studies provided two terms for abundant taxa using different thresholds, including locally abundant (> 1%) and regionally abundant (> 0.1%) taxa^45–50^, while some other studies used multiple terms to describe abundant and rare taxa, such as “always abundant taxa”, “conditional abundant taxa”, “moderate taxa”, etc.^51–54^. Considering a large geographical area was involved in this study, in which large diversification of microbial communities could be found from site to site, we used the term “regionally abundant taxa” in this study by setting the cutoff at > 0.1% of relative abundance. Using such thresholds, we uncovered that some rare taxa act as network core species in the communities, exhibiting wide adaptation across a large geographical scale. To control the positive rate in the sequence analyses, one of the effective ways is to remove ASVs with extremely low relative abundance^55^. Although the removal of rare species may change network topology, the high positive rate is more destructive to the co-occurrence network^56^. Therefore, we followed previous studies by removing ASVs with extremely low relative abundance (ASVs < 25 reads) to reduce spurious correlations^21,57–59^. Furthermore, BiSSE model predicted that rare taxa were potential candidates to become abundant under disruptive environmental events, speculating ecological perspectives of the trends in microbial assemblages in alkaline lake sediments under future environmental scenarios. These results provided important ecological values of structure, assembly, and predicted future dynamics of microbial communities in alkaline lake sediments, offered valuable microbial resources under extreme environments.

The geographical distribution of microbial communities often affected by diversification of environmental factors and ecological processes^60^, which is crucially linked to microbial structure, community assembly, and diversity variations^61,62^. The microbial distribution can be dissected by spatial decay pattern, which relate to dispersal limitations and environmental heterogeneity^63,64^. Previous studies revealed that significant distance-decay relationships in microbial communities were observed across various habitats^65–67^, often driven by diverse environmental parameters^46,68,69^. In our study, DDR analysis showed that the community structure of abundant taxa had large variations as geographical distance increased. Conversely, the structure of rare taxa remained relatively consistent regardless the geographical distance, possibly due to the broader environmental adaptation ability (Fig. 2d). Rare taxa are usually more sensitive to environmental changes than abundant ones^33^, owing to their sensitivity to deterministic processes predominantly driven by environmental selection^16,70^. Our results align with these findings, further highlighting the specific dynamics of rare taxa in microbial communities of alkaline lake sediments.

We found that pH and MAT were found as key environmental factors that influenced the assembly of abundant and rare taxa in alkaline lake sediments, respectively. While temperature was considered as a major driver on biological processes that generally affects microbial community assembly^71,72^, increasing temperatures could notably reduce the stochasticity of community assemblages and significantly alter microbial biodiversity^73–75^. Abundant taxa occupy broader niches with higher species abundance compared to rare taxa. Therefore, the influence of temperature variations on abundant taxa may have great impact in large-scale samples. For rare taxa, pH is reported as an important factor regulating communities in extreme environments, such as lake sediments and glacial soils^28,76^. This is probably because pH can directly or indirectly affect the characteristics of organic matter in sediments, influencing factors like nutrient availability, cationic metal solubility, characteristics of organic carbon, and salinity^77^. These impacts impose physiological constraints on prokaryotes, consequently affecting their metabolism^78,79^. High alkalinity environments provide a high heterogeneous and narrow niche, offering specific conditions for rare taxa to survive in extreme environments. Collectively, although both abundant and rare taxa were significantly correlated to environmental factors in alkaline sediments, rare taxa were less influenced by geographical distance, suggesting their potentials as environmental indicators monitoring large-scale responses to global climate change.

Microbial communities play distinct roles in maintaining microbial diversity and ecological stability under extreme habitats^80,81^. While abundant taxa possessed a vast majority of relative abundance in the microbial communities, rare taxa are usually found with high diversity, which link to a high spectrum of ecosystem multifunctionality and substantially provide ecological potential for adaptation under different environmental conditions^70,82,83^. Rare taxa respond to more evolutionary pressures under environmental changes than abundant taxa, and such pressures can promote the accumulation of adaptive characteristics in the evolutionary process^6,76,84^. Thus, rare taxa are considered as a species reservoir to support ecosystem stability against environmental disturbance and fluctuations. Additionally, the network analysis in our study showed that rare taxa act as network core species in the co-occurrence network, which are vital in species interactions^8,85^. Although rare taxa are less likely to be found as hubs to maintain the network topology as they are outnumbered by abundant taxa, previous studies suggested that rare species can also be critical to the network as keystone taxa^8,26,50,86^. Here, we use the term ‘network core species’ instead of ‘keystone taxa’ to avoid overstating rare taxa as keystones, as this result was only referred to co-occurrence networks. Nevertheless, our finding aligns with previous studies under same thresholds for keystone taxa (degree > 100, betweenness centrality < 5000), suggesting that rare taxa are possibly act as network core species in the co-occurrence network^12,26,50,87^. Particularly, network core species usually disproportionately maintain microbial network structure and functional stability^88,89^, such as genus *Desulfosporosinus* found in the rare taxa in our results, who was reported as a key member of sulphur reduction bacteria involved in the sulphur cycles^90,91^. Therefore, the high proportion of core taxa among rare species found in this study implied that these members may be critically involved in sustaining the ecosystem functioning in the alkaline lake sediments. Further studies such as metagenomic and metatranscriptomic analyses are required to confirm their genetic or functional capacity rather than functional predictions in the maintenance of microbial community and ecological functions.

Microbial community structure and diversity are not only influenced by immediate environmental factors, but also by historical and evolutionary events. In this context, generalists are critical in sustaining diversity of microbial species^30^. Therefore, exploring microbial communities from an evolutionary perspective can help understand the species formation and persistence of diversity. Under extreme environmental conditions such as high pH, microorganisms often experience reduced growth rates due to environmental stresses^92^. However, such harsh environments can also drive microorganisms to develop specialised survival strategies, such as reducing energy consumption, increasing biomass yield, or optimising nutrient uptake efficiency, to overcome environmental stresses^93^. In alkaline lake sediments, higher speciation rate of rare taxa suggested that new species might emerge from these rare taxa, possibly due to their better adaptability under harsh environmental conditions compared to abundant ones. Their narrow niches might confer advantages for faster reproduction and differentiation to maintain their competitiveness against abundant taxa^94^. Hence, rare taxa in alkaline lake sediments may be the source of the diversity formation, which may be able to expand microbial diversity and improve ecosystem resilience to environmental changes, ultimately promote ecosystem stability.

The dynamics of specific groups of microbial species influenced by environmental variations often result in a balance between specification and extinction^9^. These microbial groups occupy specific ecological niches and thrive or diminish under certain environmental conditions or disturbances, ensuring the resilience of microbial communities to maintain ecological functions^95,96^. In this study, we observed a higher transition rate from rare to abundant taxa than vice versa, suggesting that rare taxa could serve as a potential candidate pool to become abundant under certain environmental disturbances, or in the process of recovery and succession after destructive events. This observation was usually referred to the definition “seed banks” where rare taxa could be a genetic reservoir to overcome environmental impacts from species extinction^97^. These taxa often characterised by slow growth rates but performed well in stress resistance^98,99^. Such dynamics in response to environmental disturbances were evidenced in many habitats, such as plankton, sand, saline sediments^100–102^. Therefore, rare taxa possess a critical niche in extreme environment, including alkaline lakes, enabling ecosystems to respond and adapt to changing conditions.

**Fig. 6:**
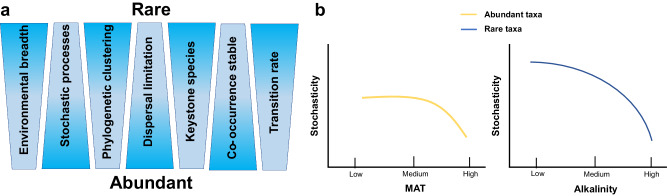
Conceptual paradigm of abundant and rare taxa alkaline lake sediment microbiota. **a** Characteristics of environmental adaptations, community assemblies, co-occurrences, and evolution patterns of abundant and rare taxa in lake sediment microbial communities. **b** Trends of stochasticity in abundant taxa under the influence of MAT, and in rare taxa under the influence of alkalinity.

In this study, we developed a conceptual paradigm to analyse environmental adaptations, ecological assembly processes, and the roles of abundant and rare taxa in the co-occurrence network, along with the evolutionary characteristics of microbial communities in alkaline lake sediments. This study spanned a vast region in China, including Qinghai-Tibet Plateau, Inner Mongolia, and Xinjiang Uygur Autonomous Region. Our findings revealed that compared to abundant taxa, rare taxa exhibited broader environmental breaths and closer phylogenetic clustering. In the process of community assembly, abundant and rare sub-communities were dominated by different processes and environment factors. Rare taxa predominantly responded to deterministic processes, while the assembly of abundant taxa was more governed by stochastic processes. Dispersal limitation was the main ecological process that revealed the importance of spatial factors affecting abundant taxa (Fig. 6a). Furthermore, pH and MAT were the primary factors that mainly mediated the assembly of abundant and rare taxa, respectively (Fig. 6b). In terms of microbial interactions, while abundant taxa crucially involved in maintaining community stability, rare taxa were critically linked to community functions and microbial interactions. Evolutionary prediction suggested that rare taxa were potential candidates to become abundant taxa. Overall, rare taxa have key significance in maintaining community stability, functional diversity, and adaptation to environmental changes from an evolutionary perspective. These findings provide a new perspective for the ecological significance of microbial communities in alkaline lake sediments, highlighting the importance of microbial diversity in ecosystem resilience and function.

## Methods

### Sample collection

Lake sediment samples were collected at late summer to autumn season, from July 2020 to October 2021. A total of 32 alkaline lakes were investigated, including 19 lakes in Qinghai (Qinghai-Tibet Plateau) collected at late July 2021, 7 lakes in Inner Mongolia collected at early September 2021, and 6 lakes in Xinjiang Uygur Autonomous Region collected at early October 2020, resulting in 136 lake sediment samples (details of sample information in Supplementary Fig. 1 and Supplementary Table 1). For each lake, 4–5 sampling sites (*n* = 4 or 5) were randomly selected, with each two sampling sites at least 50 m apart. At each sampling site, lake sediment was taken at 0–10 cm depth 5 times using a sterile spatula within 1 m^2^ area before mixed thoroughly to form a composite sample. Each composite sample was separated into two parts: about 5 g sediment sample was transferred in a 2 mL sterile cryogenic tube and immediately frozen in liquid nitrogen for DNA extraction; and the other part of sediment (~100 g) was collected in a sterile zip lock bag for physiochemical measurement. All samples were transported to laboratory on dry ice and store at −80 °C (for DNA extraction) or −20 °C (for physiochemical measurement) until further analysis.

### Measurement of environmental parameters

For geographical parameters, mean annual temperature (MAT) and mean annual precipitation (MAP) data of each lake were obtained from the WorldClim database (https://www.worldclim.org). For measurement of physicochemical properties, sediment samples were freeze-dried in a freeze-drying machine (SCIENTZ-10N, SCIENTZ, Ningbo, China), ground and sieved through a 10 mm sieve. Approximately 2 g freeze-dried sediment sample was dissolved in 20 mL sterile water in a 50 mL falcon tube, shaking vigorously to mix thoroughly. The pellet was precipitated by centrifugation at 5,000 g for 5 min, and the supernatant was collected for further measurement. pH and electrical conductivity (EC) were measured by portable water quality multi-parameter analyzer (Multi3430, WTW, German); Lithium (Li^+^), sodium (Na^+^), ammonium (NH_4_^+^), potassium (K^+^), magnesium (Mg^2+^), calcium (Ca^2+^), fluorine (F^-^), chlorine (Cl^-^), nitrite (NO_2_^-^), bromine (Br^-^), nitrate (NO_3_^-^), and sulfate (SO_4_^2-^) were measured by ion chromatography (SH-CC-3L, SHINE, Qingdao, China). Details of sampling sites as well as physical and chemical parameters were recorded in Supplementary Table 1.

### DNA extraction and amplicon sequencing

Total DNA was extracted from 0.5 g fresh sediment samples using DNeasy PowerSoil Pro Kit (Qiagen, German) following the manufacturer’s instructions. DNA quantity and purity were measured using NanoDrop 2000 Spectrophotometer (Thermo Scientific, MA, USA) and Qubit (Thermo Scientific, MA, USA), respectively. Amplicon sequencing was performed targeting the hypervariable V4 region of the 16S rRNA gene using the primers 515 F (5’-GTGYCAGCMGCCGCGGTAA-3’) and 806 R (5’-GGACTACHVGGGTWTCTAAT-3’)^103^. Library preparation and amplicon sequencing was conducted on an Illumina HiSeq 2500 platform with paired-end 150 chemistry at Novogene Bioinformatics Technology Co., Ltd., Tianjin, China.

### Sequences processing

The raw paired-end FASTQ sequences were processed using QIIME2 pipeline (v2021.2) for the based microbiota analyses^104^. DADA2 was used for denoising and quality filtering before constructing the feature table at 100% sequence identity^105^. Phylogenetic tree of the aligned sequences was constructed using FastTree 2, and sequences were clustered at 100% identity into amplicon sequence variants (ASVs) using deblur^106^. Taxonomic classification of ASVs were conducted against a pre-trained Naïve Bayes taxonomy classifier based on the SILVA reference database (release 138)^107^. To reduce the classification of rare taxa affected by accidental factors, ASVs with fewer than 25 reads were removed. For subsequent analysis, the ‘trimmed means of M’ (TMM) method in the R package ‘edgeR’^108^ was used to rarefy the filtered ASV sequence counts^109^. Functional prediction based on ASVs were performed using PICRUSt2^110^ and Tax4Fun2^111^.

### Analysis of abundant and rare taxa

To investigate the differences of ecological strategies of subcommunities in alkaline lake sediments, ASVs were separated into three groups in terms of their relative abundances: abundant taxa (≥ 0.1%), rare taxa (≤ 0.001%), and intermediate taxa (between 0.001% and 0.1%), following the method reported in previous studies^21,47,50^. Based on the above definitions, abundant and rare taxa may have some overlaps, that is, some ASVs may be rare in one site and abundant in another. However, it is consistent with the fact that many ASVs are conditionally rare or abundant taxa^46,112^.

### Phylogenetic turnover and environmental breath analyses

The β-diversity was estimated as β-mean nearest taxon distance matrix (β-MNTD) reflecting the pairwise phylogenetic turnover between communities, which was calculated using the ‘comdistnt’ function in the R package ‘picante’^113^. The standardized index employing the mean nearest-taxon distance (SES.MNTD) was determined using null model ‘taxa.labels’ with 1000 iterations to assess the phylogenetic clustering of abundant and rare taxa. When the SES.MNTD value < 0, lower values indicate that the phylogeny is more tended to be clustering, that is, co-occurring species are more closely related than expected by chance. When the value > 0, higher values indicate more dispersed phylogeny, that is, occurring species are less closely related than expected by chance. The distance decay relationship (DDR) was used to calculate the slope of an ordinary least-squares regression of Bray-Curtis similarity and phylogenetic beta diversity change with geographic distance, following the method described in a previous study^114^. Geographic distance was calculated using the ‘distm’ function in the R package ‘geosphere’^115^. Canonical correspondence analysis (CCA) was performed using R package ‘vegan’ to assess community composition in response to environmental factors^116^. Principal coordinates of the neighbourhood matrix (PCNM) were calculated using the ‘PCNM’ function in the R package ‘vegan’. In CCA, forward selection was constructed to select environmental factors that were statistically significant (*P* < 0.05). Variance partition analysis (VPA) was performed using the function ‘varpart’ in the R package ‘vegan’ to explain the differences in community composition by physical and chemical factors and geographical distance. Random forest (RF) analysis was used to explore the importance of environmental factors on β-nearest taxon index (β-NTI) dimension reduction data using R package ‘randomForest’. Principal Component Analysis (PCA) was performed using R package ‘gmodels’ to select PC1 axis data as β-NTI dimension reduction data^117^.

To measure the difference of threshold values between abundant and rare taxa in various of physicochemical factors, a threshold indicator taxa analysis (TITAN) was performed using the R package ‘TITAN2’^118^. TITAN distinguishes taxa by positive values (Z +) and negative values (Z−). Z + taxa increase in abundance and frequency after the point of change, while Z − taxa exhibit the opposite pattern. Sums of indicator taxa scores (z-scores) for ASVs was used to determine upper (sum (Z +)) and lower (sum (Z−)) threshold based on environmental variables to reflect the range of potential change thresholds in abundant and rare taxa.

### Quantification of ecological modelling

The null model quantifying the potential contribution of stochastic and deterministic processes of abundant and rare taxa was performed following the framework constructed by Stegen et al.^119^. The relative contribution of different ecological processes (dispersal limitation, homogeneous dispersal, homogeneous selection, variable selection, and undominant fraction) in the assembly of abundant and rare taxa were assessed based on the inferred Community Assembly Mechanisms by Phylogenetic-bin (iCAMP)^120^. The variation in taxonomic and phylogenetic diversities were calculated according to the Bray-Curtis-based Raup-Crick (RC_bray_) and β-NTI. Homogeneous dispersal is defined when |β-NTI| < 2 and RC_bray_ < –0.95, dispersal limitation is defined when RC_bray_ > 0.95, while undominated fraction (*i.e*., weak selection, ecological drift, diversification, weak dispersal) is defined when |β-NTI| < 2 and |RC_bray_| < 0.95.

Mantel test, partial Mantel test, and the measurement of the relationship between β-NTI and the derived physicochemical factors along with statistical analysis of the comparisons (999 permutations) were determined using R package ‘vegan’. Linear regression models were applied to assess the correlation between β-NTI and physicochemical factors that drive the construction of abundant and rare communities.

### Co-occurrence network analysis

Network analysis based on Spearman’s correlation was performed using R package ‘igraph’ to explore the cooccurrence patterns of abundant, intermediate, and rare taxa^121^. To reduce the complexity of the datasets, ASVs were screened at frequency higher than 8 across all samples to construct a co-occurrence network. A credible co-occurrence event was considered when a correlation between taxa with the Spearman’s correlation coefficient above 0.6 and Benjamini & Hochberg corrected (BH-corrected) *P*-value below 0.01^50,85^. To describe the network topology, four unique node-level topological features (degree, betweenness, closeness and eigenvector centrality) were calculated in the R package ‘igraph’. Statistical analysis in measured node-level attributes across different taxa was conducted using Wilcoxon test. In addition, nodes with degree above 100 and betweenness centrality values below 5000 were recognized as network core species in co-occurrence networks^23^. Network visualization and modular analysis were performed in Gephi (v 0.9.2)^122^.

### Evolutionary trends of abundant and rare taxa

The binary-state speciation and extinction (BiSSE)^123^ model was used to predict the evolutionary traits and ecological roles of abundant, intermediate, and rare taxa. This model considers abundant, intermediate, and rare taxa as distinct evolutionary states, calculating speciation rates, extinction rates, and state-transition rates. BiSSE model was performed with the R package ‘diversitree’^124^. Phylogenetic trees of abundant, intermediate, and rare taxa as key parameter inputs to the BiSSE model were established using function ‘match.phylo.data’ in R package ‘picante’. Linearization and reconstruction of the trees into an ultrametric tree was conducted using function ‘chronoMPL’ in R package ‘ape’. The rate parameters were estimated with a maximum likelihood (ML) method using function ‘starting.point.bisse’ to obtain a sensible starting point, with 10,000-step Markov chain Monte Carlo (MCMC) simulations performed to ensure the stability of the final estimate.

### Reporting summary

Further information on research design is available in the Nature Research Reporting Summary linked to this article.

### Supplementary information

Supplementary information

Reporting summary

## Data Availability

The raw amplicon sequences are available in the CNCB-NGDC database (https://ngdc.cncb.ac.cn/) under project PRJCA022538 and NCBI SRA under project PRJNA1090246.
