# Supplementary material for: Large scale exploration reveals rare taxa crucially shape microbial assembly in alkaline lake sediments
Source: NPJ Biofilms Microbiomes. 2024 Jul 28;10:62. doi: 10.1038/s41522-024-00537-1 (PMC11284227; doi:10.1038/s41522-024-00537-1)
Supplement: Supplementary file 1 — Supplementary information [file 41522_2024_537_MOESM1_ESM.pdf]

Supplementary information

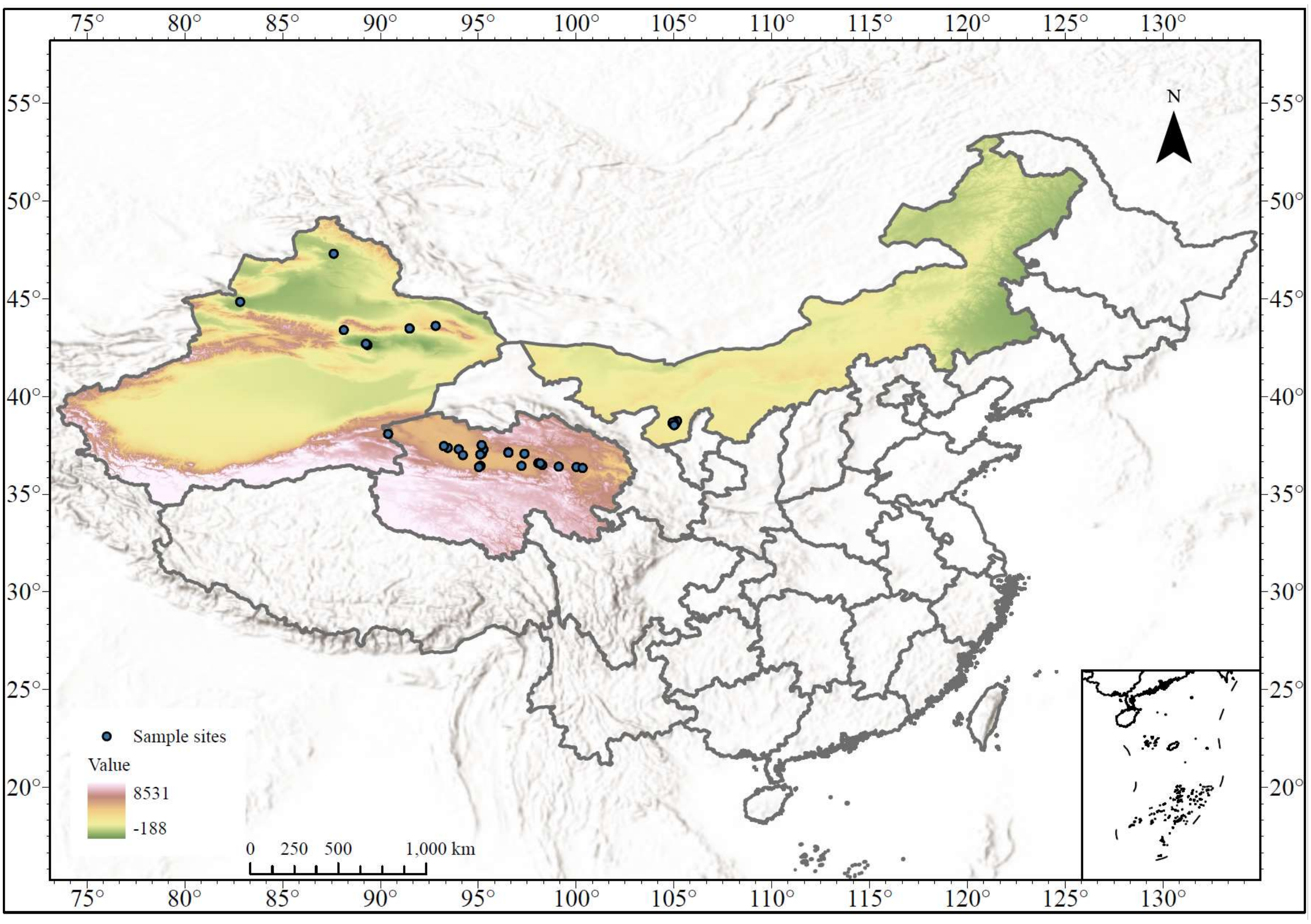

Supplementary Fig. 1. Sampling map in west-northwest China.

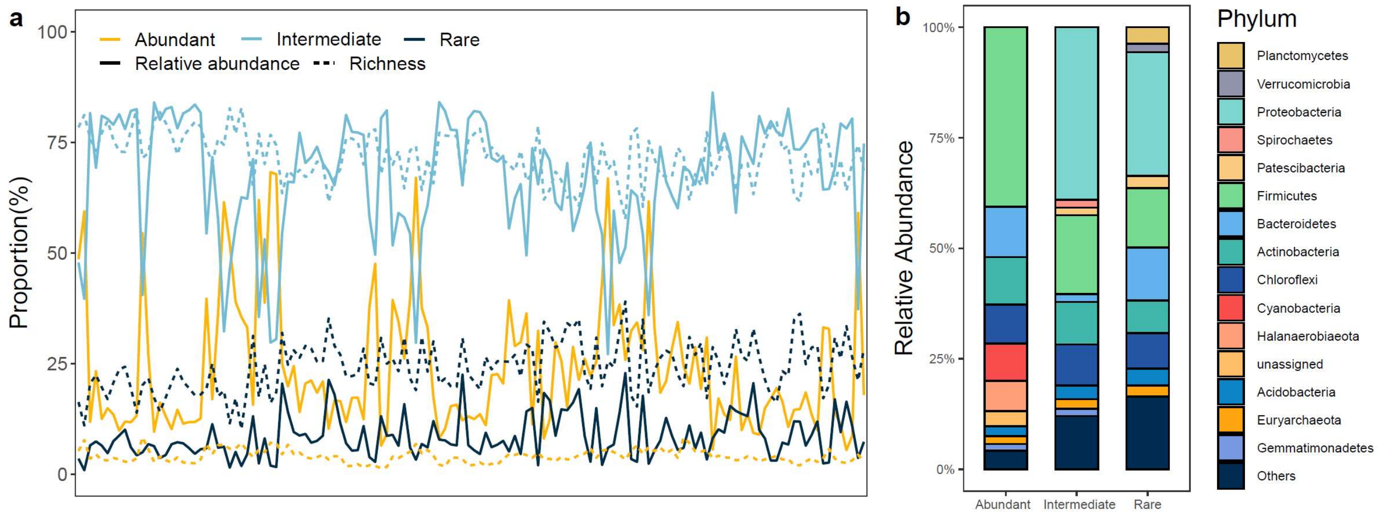

Supplementary Fig. 2. Distribution patterns of abundant and rare microbial communities in alkaline lake sediments. **a** The proportion of the OTU richness and relative abundance of abundant, intermediate, and rare taxa across the 136 alkaline lake sediment samples. **b** Relative abundances of the top 10 phyla in abundant, intermediate, and rare microbial communities.

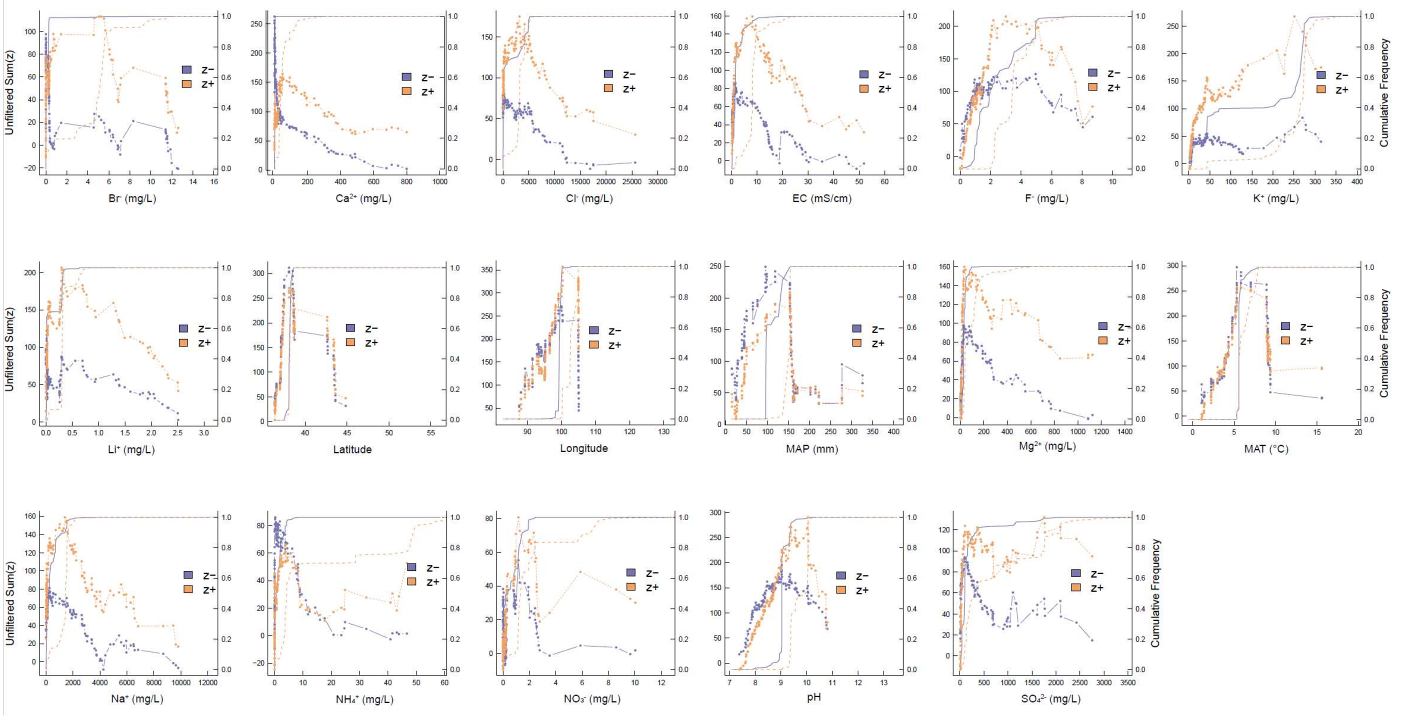

Supplementary Fig. 3. Occurrence thresholds of abundant taxa in alkaline lake sediments in response to environmental parameters on the community composition and structure. The community members were normalised by z-score showing the decline of taxa with the increase of environmental gradient (z-, purple), and the increase of taxa with the increase of environmental gradient (z+, orange).

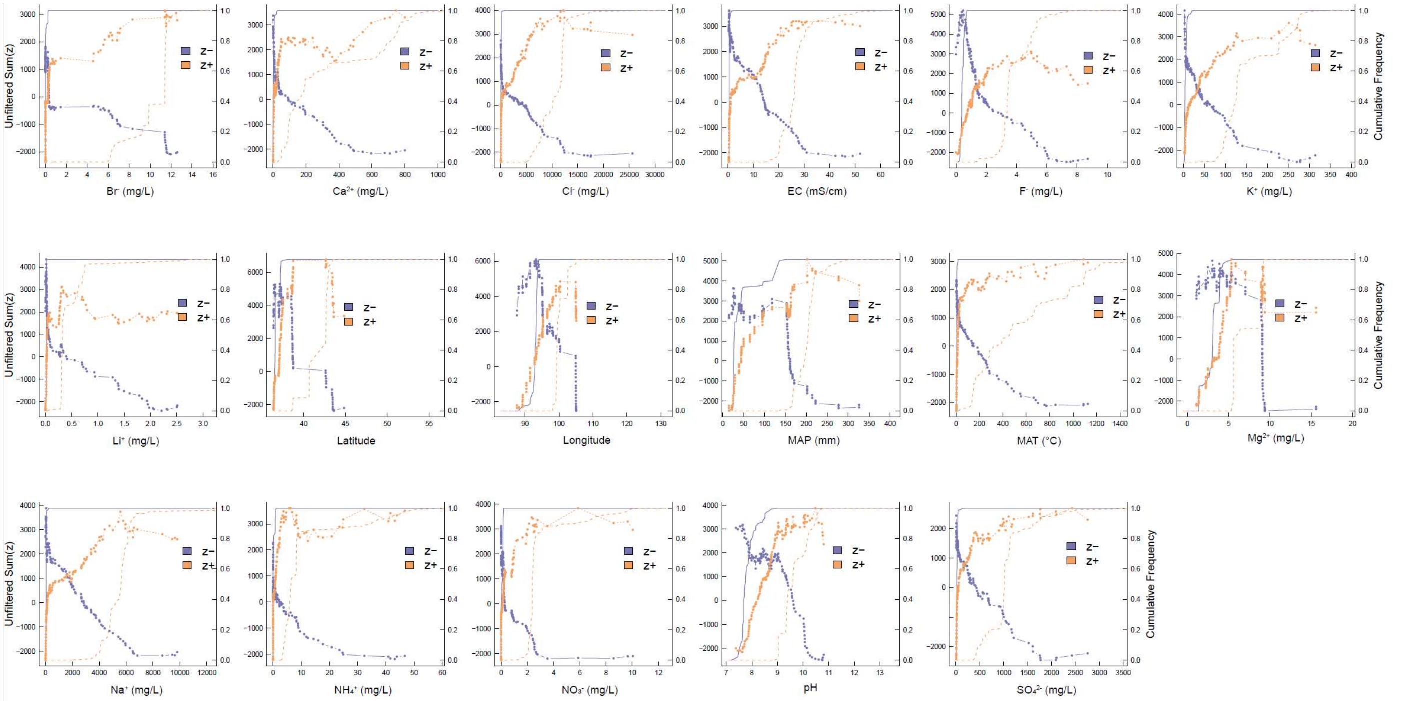

Supplementary Fig. 4. Occurrence thresholds of rare taxa in alkaline lake sediments in response to environmental parameters on the community composition and structure. The community members were normalised by z-score showing the decline of taxa with the increase of environmental gradient (z-, purple), and the increase of taxa with the increase of environmental gradient (z+, orange).

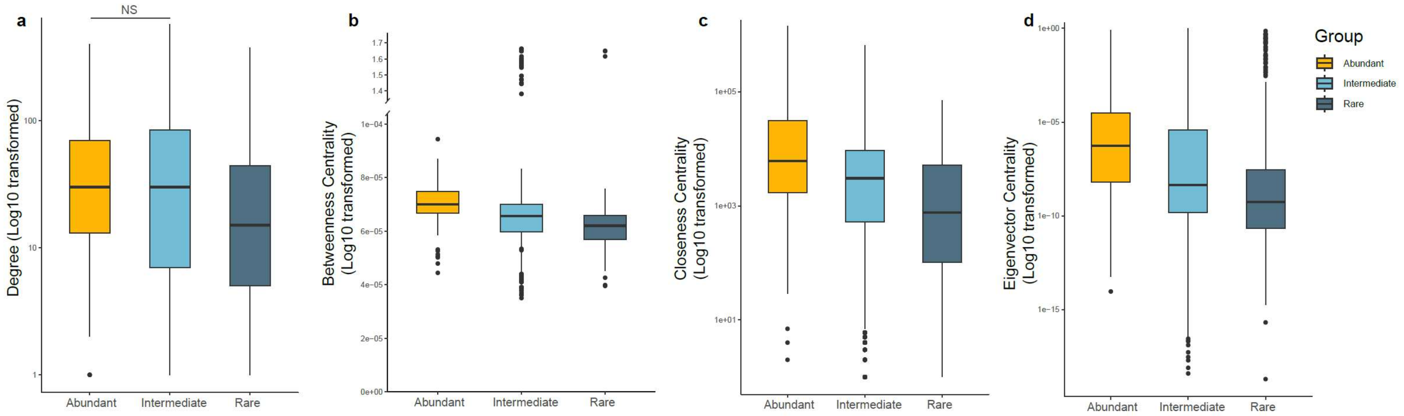

Supplementary Fig. 5. Comparison of node-level topological characteristics of abundant (yellow), intermediate (cyan), and rare taxa (navy), including **a** degree (Log10 transformed), **b** closeness centrality (Log10 transformed), **c** betweenness centrality (Log10 transformed), and **d** eigenvector centrality (Log10 transformed). Significant differences (P < 0.05) were found across all groups except abundant and intermediate taxa in degrees. The boxplot shows the distribution of data, the central dot in the box represents the median, the box bounds represent the 25th and 75th percentiles, and whiskers represent the minima to maxima values.

Supplementary Table 1. Geological parameters and physiochemical parameters of samples in this study.The unit for anions and cations: mg/L, electic conductivity (EC): ms/cm, mean annual temperature (MAT): °C, mean annual precipitation (MAP): mm.

SampleID	Location	Year	Region	Longitude	Latitude	pH	EC	Li^+	Na^+	NH4$^+$	K^+	Mg^{2+}	Ca^{2+}	F^-	Cl^-	NO_2^-	Br^-	NO_3^-	SO_4^{2-}	MAT	MAP
XJ_S147	WLGH	2020	Xinjiang	87.60858	47.29811	10.77	15.93	0.278	3743.29	0	300.868	19.798	0	4.4	3207.46	0	6.054	0	1756.03	9.07083	163
XJ_S148	WLGH	2020	Xinjiang	87.60858	47.29811	11.25	11.58	0.256	2671.72	0	278.284	5.54	0	7.734	2221.58	0	6.214	0.984	709.944	8.92083	161
XJ_S149	WLGH	2020	Xinjiang	87.60858	47.29811	10.05	0.441	0.0203	54.4421	2.2539	13.7861	8.343	1.6522	5.8302	54.5476	0.0692	0.2957	0.206	14.2324	9.1125	156
XJ_S113	ABH	2020	Xinjiang	82.82485	44.83819	10.35	1.006	0.0228	151.643	0.2065	32.0254	6.3351	0.6043	5.3479	165.623	0	0.4622	0.1966	34.3976	9.1125	156
XJ_S114	ABH	2020	Xinjiang	82.82485	44.83819	9.57	0.1705	0.016	6.8361	1.3692	9.8067	4.8145	7.0719	2.7815	5.619	0.0422	0.2276	0.199	3.7982	9.1125	156
XJ_S115	ABH	2020	Xinjiang	82.82485	44.83819	9.2	0.1405	0.0174	20.5516	2.2014	15.5652	9.6531	26.5198	5.9059	18.0136	0.0837	0.2554	0.1816	9.8824	9.1125	156
XJ_S137	BLK	2020	Xinjiang	92.81924	43.6094	9.4	0.298	0.0171	6.2717	2.4062	4.492	8.1268	32.7727	1.2776	4.8137	0.2021	0.317	0.2646	13.7877	8.96667	156
XJ_S138	BLK	2020	Xinjiang	92.81924	43.6094	9.42	0.1999	0.0158	3.4068	0.3724	3.2917	5.4008	23.7307	0.9733	1.9708	0.0692	0.2288	0.2375	6.5062	8.96667	156
XJ_S139	BLK	2020	Xinjiang	92.81924	43.6094	10.27	0.1972	0.0164	3.1745	0.3575	3.7049	5.1193	24.8467	0.9831	1.7813	0.0789	0.2359	0.1945	7.7332	8.96667	156
XJ_S140	BLK	2020	Xinjiang	92.81924	43.6094	9.1	0.469	0.0266	24.769	4.6676	17.552	22.334	26.9463	3.7353	11.5057	0.0898	0.269	0.2096	11.9921	9.10417	158
XJ_S180	QJJ1	2020	Xinjiang	91.48069	43.48016	9.63	0.845	0.0279	129.197	8.2562	31.6378	14.827	14.3923	4.9697	54.1341	0.082	0.8528	0.249	27.3099	9.10417	158
XJ_S183	QJJ1	2020	Xinjiang	91.48069	43.48016	10.48	13.75	0	3388.28	0	226.928	18.1	0	2.41	2113.24	0	5.66	0	2091.09	9.07083	163
XJ_S186	QJJ1	2020	Xinjiang	91.48069	43.48016	11.05	13.28	0.268	3086.83	0	418.96	14.638	0	13.616	2337.92	4.724	6.906	0.954	980.196	9.10417	158
XJ_S189	QJJ1	2020	Xinjiang	91.48069	43.48016	9.53	1.069	0.0259	177.697	2.7665	40.6743	11.7861	5.4407	9.1784	105.807	0.2167	0.3372	0.2183	85.9486	9.10417	158
XJ_S171	QJJ2	2020	Xinjiang	91.49148	43.46783	9.2	0.232	0.0255	5.8799	1.1008	19.8701	9.9357	9.3246	0.9864	3.5214	0.185	0.2319	0.3023	6.5569	9.07083	154
XJ_S174	QJJ2	2020	Xinjiang	91.49148	43.46783	10.08	0.333	0.0287	31.792	0.9387	19.8535	7.0812	10.4128	1.0962	28.3603	0.1144	0.2528	0.301	7.6562	9.07083	154
XJ_S177	QJJ2	2020	Xinjiang	91.49148	43.46783	10.76	0.945	0.0304	163.004	2.0843	39.0906	14.3672	0.816	6.6705	102.857	0.0608	0.3759	0.2133	25.2558	9.07083	154
XJ_S128	DBC	2020	Xinjiang	88.12314	43.398	9.61	0.972	0.0293	168.81	0.1421	38.7685	12.1628	0.737	6.0336	110.37	0.212	0.3621	0.26	37.6541	9.07083	154
XJ_S129	DBC	2020	Xinjiang	88.12314	43.398	10.15	0.608	0.0268	51.5459	2.3223	38.7646	31.8819	1.6329	10.9949	42.4779	0.1539	0.3266	0.2755	12.6778	9.07083	161
XJ_S130	DBC	2020	Xinjiang	88.12314	43.398	10.07	0.543	0.0244	50.6566	2.2262	34.5843	25.2273	3.0089	9.471	31.6163	0.1408	0.3004	0.2224	17.038	9.07083	161
XJ_S100	ADH	2020	Xinjiang	89.25483	42.69185	10.06	0.366	0.0274	29.8326	0.4986	26.4076	14.8929	3.7942	5.1235	22.415	0.0508	0.0942	0.1898	11.9909	9.07083	161
XJ_S97	ADH	2020	Xinjiang	89.25483	42.69185	10.07	0.434	0.0372	26.4478	0.5415	30.1878	27.4215	1.6145	7.2924	17.4405	0.0277	0.0277	0	2.7242	9.07083	161
XJ_S98	ADH	2020	Xinjiang	89.25483	42.69185	10.88	18.9	0.28	4403.39	0	467.562	2.942	0	1.322	4869.33	0	7.152	0	1607.61	9.07083	163
XJ_S99	ADH	2020	Xinjiang	89.25483	42.69185	9.71	2.68	0.0523	400.566	0	86.5916	26.3585	2.6782	9.2341	443.291	0	0.7949	0.0451	259.604	9.07083	163
XJ_S106	ADH2	2020	Xinjiang	89.33286	42.6302	10.74	13.48	0.26	2971.89	0	400.192	13.194	0	6.11	3501.46	0	6.838	0	1002.45	9.07083	163
XJ_S107	ADH2	2020	Xinjiang	89.33286	42.6302	10.61	15.15	0.256	3393.35	0	441.034	14.056	0	7.562	3888.86	0	7.082	0	1174.36	9.07083	163
XJ_S108	ADH2	2020	Xinjiang	89.33286	42.6302	10.81	12.56	0.264	2891.86	0	314.052	6.916	0	5.352	2566.04	0	6.838	0.924	685.292	8.92083	161
NM_K7	TGNE	2021	Inner Mongolia	105.1571	38.74749	11.03	10.1	0	2247.86	0	277.806	6.64	0	3.656	2258.25	0	6.398	1.166	463.87	8.92083	161
NM_K8	TGNE	2021	Inner Mongolia	105.1571	38.74749	10.82	19.56	0.294	4765.04	0	548.58	2.508	0	8.058	4199.9	0	8.316	0.982	1211.51	8.92083	161
NM_K9	TGNE	2021	Inner Mongolia	105.1571	38.74749	7.887	23.8	2.6	4004.91	0	106.122	770.074	459.346	0.62	9125.83	0	0	0	584.824	5.17083	29
NM_K10	TGNE	2021	Inner Mongolia	105.1571	38.74749	8.045	30.9	1.84	6281.33	0	98.295	428.575	383.155	0.945	14045.6	24.185	0	2.135	651.21	5.17083	29
NM_K18	ZEQK	2021	Inner Mongolia	104.9947	38.68701	7.708	12.74	2.326	2650.17	0	62.564	76.224	49.132	1.508	4744.62	0	0	0	203.924	4.32917	27
NM_K19	ZEQK	2021	Inner Mongolia	104.9947	38.68701	7.782	9.55	1.786	1902.25	0	52.768	58.912	30.542	1.286	3421.07	0	0	0	157.884	4.32917	27
NM_K20	ZEQK	2021	Inner Mongolia	104.9947	38.68701	7.814	11.16	2.212	2201.44	0	55.704	90.872	74.162	1.506	4496.21	12.978	0	0	294.734	4.32917	27
NM_K21	ZEQK	2021	Inner Mongolia	104.9947	38.68701	8.318	8.22	1.496	1589.52	1.018	44.66	48.214	35.77	1.342	3253.07	5.978	0	0.85	159.906	4.32917	27
NM_K22	BGDWS	2021	Inner Mongolia	104.9437	38.67128	9.563	0.432	0.0878	70.5882	0.0635	6.394	5.9776	8.4065	1.0726	131.923	0.1817	0.2314	0.1863	19.0521	4.23333	22
NM_K23	BGDWS	2021	Inner Mongolia	104.9437	38.67128	9.274	0.726	0.0844	60.7558	0	8.4405	17.9956	31.774	0.7191	97.7721	0.0748	0	0.203	84.3337	4.23333	22
NM_K24	BGDWS	2021	Inner Mongolia	104.9437	38.67128	9.012	0.628	0.0691	71.903	0.0734	7.5257	7.6292	15.785	0.6769	138.211	0	0.2287	0.1868	32.7686	4.23333	22
NM_K25	BGDWS	2021	Inner Mongolia	104.9437	38.67128	9.654	2.93	0.0657	497.672	5.6213	5.3561	3.3624	9.5407	1.4558	902.246	0	0.2296	0.1796	22.1109	4.23333	22
NM_K11	GS	2021	Inner Mongolia	104.9695	38.65173	10.05	3.45	0.2061	535.121	8.9917	15.6503	17.5606	32.0764	1.0799	1001.21	0	0.277	0.174	73.1848	3.86667	25
NM_K12	GS	2021	Inner Mongolia	104.9695	38.65173	8.465	1.742	0.1408	366.594	8.2729	9.3387	8.0069	15.2962	1.0588	681.37	0	0.2653	0.3113	31.8631	3.86667	25

NM_K13	GS	2021	Inner Mongolia	104.9695	38.65173	9.365	27.7	1.395	6490.72	0	111.62	149.56	106.22	0.985	12323.5	0	0	10.04	103.62	3.86667	25
NM_K14	GS	2021	Inner Mongolia	104.9695	38.65173	8.383	26.8	1.33	5783.7	0	103.245	139.36	92.835	0.895	11046.6	0	0	0	97.62	3.86667	25
NM_K15	NE	2021	Inner Mongolia	104.9311	38.62513	10.025	1.846	0.0502	188.461	0.5509	6.7728	53.0357	55.49	1.0554	410.384	0.704	0.2931	0.1395	164.243	1.36667	65
NM_K16	NE	2021	Inner Mongolia	104.9311	38.62513	9.878	0.439	0.0204	36.4222	0.4737	1.9666	9.5195	20.7402	0.3677	74.6139	0.2946	0	0.1697	33.0493	1.36667	65
NM_K17	NE	2021	Inner Mongolia	104.9311	38.62513	10.19	0.956	0.0524	91.2283	1.0177	8.6307	25.6796	36.5239	0.88	109.986	0.0485	0.2649	0.3663	89.6061	1.36667	65
NM_K1	ABTEK	2021	Inner Mongolia	105.0799	38.61763	8.767	0.84	0.0515	77.7687	7.6408	6.8689	32.4943	12.6101	1.2437	94.509	0	0.3906	0.1023	27.9775	1.36667	65
NM_K2	ABTEK	2021	Inner Mongolia	105.0799	38.61763	9.176	0.877	0.0419	117.525	11.7845	7.8899	18.4353	15.1882	0	176.592	0	0	0	9.1772	2.24167	326
NM_K3	ABTEK	2021	Inner Mongolia	105.0799	38.61763	9.343	0.556	0.0407	60.4786	6.7555	7.6807	15.3097	7.8351	0.1653	72.9743	0.0778	0.3305	0.1149	35.2043	2.24167	326
NM_K4	ABTEK	2021	Inner Mongolia	105.0799	38.61763	9.043	0.367	0.0255	4.5644	8.8834	6.3071	9.1325	47.078	0.2489	7.7796	0.0722	0.2792	0.0947	25.9741	2.24167	326
NM_K5	ABTEK	2021	Inner Mongolia	105.0799	38.61763	8.976	0.45	0.0241	37.8153	3.813	6.0854	10.0416	22.4725	0.3919	65.8823	0.0562	0.2525	0.1183	17.0179	2.47917	367
NM_K6	ABTEK	2021	Inner Mongolia	105.0799	38.61763	8.647	1.587	0.0789	122.439	24.8638	22.0108	75.7441	31.9235	0.3855	164.141	0	1.4495	24.622	50.8924	2.47917	367
NM_K26	NRYG	2021	Inner Mongolia	105.0131	38.5308	9.034	0.404	0.0181	6.8413	9.5402	4.2462	10.0405	44.7261	0.2361	7.1583	0.0688	0.3263	0.0712	40.1576	2.47917	367
NM_K27	NRYG	2021	Inner Mongolia	105.0131	38.5308	10.023	0.855	0.0387	115.79	8.8365	9.8628	15.0379	6.0972	0.2008	154.057	0	0.3454	0.0454	31.1116	2.47917	367
NM_K28	NRYG	2021	Inner Mongolia	105.0131	38.5308	7.323	53.7	0.945	22015	0	250.93	1088.62	303.585	1.415	40432.4	0	11.865	0	929.89	-0.4583	277
NM_K29	NRYG	2021	Inner Mongolia	105.0131	38.5308	7.897	75.2	0.93	16729.3	149.79	228.13	1125.59	343.68	1.265	31941.7	0	18.095	1.895	1061.82	-0.4583	277
QH_S118	GSKL	2021	Qinghai-Tibet Plateau	90.39	38.08	8.321	44.9	0.79	8690.18	69.27	140.295	700.055	371.94	1.83	16905	0	11.435	0	972.325	-0.4583	277
QH_S119	GSKL	2021	Qinghai-Tibet Plateau	90.39	38.08	7.54	29.4	0.77	3405.69	0	129.025	680.6	302.305	1.045	8049.27	0	11.54	0	694.13	-0.4583	277
QH_S120	GSKL	2021	Qinghai-Tibet Plateau	90.39	38.08	7.617	22.8	0.41	4029.67	32.36	93.504	548.396	747.184	3.086	8143.62	0	6.064	0.88	0	2.22917	202
QH_S121	GSKL	2021	Qinghai-Tibet Plateau	90.39	38.08	7.65	16.25	0.342	3055.01	0	49.908	229.74	358.274	1.942	5808.05	0	0	0	682.258	2.22917	202
QH_S89	DCD	2021	Qinghai-Tibet Plateau	95.17	37.51	7.873	27.9	0.7	5961.32	0	67.49	441.12	489.52	2.085	11590.6	0	0	1.925	901.385	2.22917	202
QH_S90	DCD	2021	Qinghai-Tibet Plateau	95.17	37.51	7.452	20.7	0.312	3756.4	40.89	38.532	268.43	906.012	1.244	7063.03	0	4.55	0.858	0	2.22917	202
QH_S91	DCD	2021	Qinghai-Tibet Plateau	95.17	37.51	8.152	0.804	0.0228	106.041	2.2238	4.5578	7.5902	27.2468	1.9197	161.523	0	0.2482	0.1999	61.3371	1.09167	222
QH_S92	DCD	2021	Qinghai-Tibet Plateau	95.17	37.51	8.34	4.09	0.0592	710.708	20.8	24.0815	24.0815	35.7285	2.9913	1136.97	0	0.7043	0.0509	99.1012	1.09167	222
QH_S110	XT	2021	Qinghai-Tibet Plateau	93.24	37.46	8.542	3.45	0.0564	499.117	24.631	30.77	67.8425	24.2946	4.9506	872.202	0	0.9711	25.5654	18.3125	1.09167	222
QH_S111	XT	2021	Qinghai-Tibet Plateau	93.24	37.46	8.69	42.2	1.95	5420.67	0	209.225	2790.28	486.29	1.825	17504.4	0	15.275	0	1522.15	1.1625	214
QH_S112	XT	2021	Qinghai-Tibet Plateau	93.24	37.46	8.454	29	1.975	2040.34	0	81.605	2725.56	933.085	1.895	12139.3	0	17.61	0	1612.58	1.1625	214
QH_S113	XT	2021	Qinghai-Tibet Plateau	93.24	37.46	8.682	6.02	0.29	1024.01	16.008	34.964	96.446	121.978	1.404	1801.01	0.668	0	0.874	360.858	3.80833	153
QH_S106	YH	2021	Qinghai-Tibet Plateau	93.44	37.37	8.543	7.57	0.296	1410.65	41.584	42.97	108.092	11.26	1.922	2581.68	0	4.61	0.882	171.122	3.80833	153
QH_S107	YH	2021	Qinghai-Tibet Plateau	93.44	37.37	8.735	8.19	0.306	1531.97	8.304	36.27	147.512	26.32	2.076	2790.81	0	0	0	328.188	3.80833	153
QH_S108	YH	2021	Qinghai-Tibet Plateau	93.44	37.37	9.346	3.4	0.0371	442.132	17.6635	14.979	83.4239	34.4661	0.6759	1135.33	0	0.5099	0.115	60.5727	3.0125	98
QH_S109	YH	2021	Qinghai-Tibet Plateau	93.44	37.37	9.578	3.79	0.0399	563.104	23.5321	17.0303	96.1055	48.9195	0.6358	1175.23	0	0.6637	0.1016	23.9404	3.0125	98
QH_S102	DT	2021	Qinghai-Tibet Plateau	94	37.3	9.325	1.433	0.0242	177.917	3.76	7.3053	26.9585	22.5139	0.7246	386.321	0	0.2509	0.1297	42.1373	3.0125	98
QH_S103	DT	2021	Qinghai-Tibet Plateau	94	37.3	8.907	2.43	0.0308	310.634	6.2085	9.798	45.8484	28.723	0.637	723.911	0	0.2879	0.1189	34.9876	3.0125	98
QH_S104	DT	2021	Qinghai-Tibet Plateau	94	37.3	8.87	0.436	0.0186	56.5132	0.4235	3.4239	7.2255	3.072	0.1099	86.517	0	0	0.2882	21.4558	3.25833	95
QH_S105	DT	2021	Qinghai-Tibet Plateau	94	37.3	9.032	0.64	0.0198	84.1789	0.7016	4.6619	11.0555	3.1685	0.1163	140.17	0.0532	0.2322	0.289	37.9424	3.25833	95
QH_S84	XCD	2021	Qinghai-Tibet Plateau	95.26	37.29	9.326	0.45	0.0193	51.5637	0.9799	3.7046	10.3891	6.2329	0.1093	96.9725	0.252	0.2577	0.1877	29.7744	3.25833	95
QH_S85	XCD	2021	Qinghai-Tibet Plateau	95.26	37.29	8.847	0.874	0.0299	101.722	0.1479	6.0995	21.3869	7.2464	0.46	195.731	0.057	0.2372	0.1417	48.2401	3.25833	95
QH_S86	XCD	2021	Qinghai-Tibet Plateau	95.26	37.29	8.905	0.222	0.0195	22.2557	0.1677	4.3261	5.4744	4.1075	0.2053	27.107	0.0449	0	0.2149	9.4838	3.25833	95
QH_S87	XCD	2021	Qinghai-Tibet Plateau	95.26	37.29	9.324	0.57	0.0228	30.7899	2.2705	6.6442	17.3824	36.3338	0.2835	37.3466	0.1199	0.2278	0.2438	77.3702	3.25833	95
QH_S88	XCD	2021	Qinghai-Tibet Plateau	95.26	37.29	8.768	0.415	0.0173	48.688	0.6986	1.543	5.075	19.8162	0.2554	97.6679	0.0497	0	0.0991	15.0289	4.81667	118
QH_S53	KLK	2021	Qinghai-Tibet Plateau	96.54	37.15	8.632	0.17	0.016	21.298	1.488	2.5649	1.5796	6.8647	0.3834	24.4324	0.0958	0	0.0968	7.0467	4.81667	118
QH_S54	KLK	2021	Qinghai-Tibet Plateau	96.54	37.15	8.732	0.366	0.0167	52.0949	0.8301	2.6136	3.5853	9.8256	0.2299	88.2033	0.1172	0	0.0941	7.199	4.81667	118
QH_S55	KLK	2021	Qinghai-Tibet Plateau	96.54	37.15	8.019	25	0.72	5013.34	55.75	119.75	349.34	133.41	0	11540.7	21.44	0	27.02	292.315	5.32083	45
QH_S56	KLK	2021	Qinghai-Tibet Plateau	96.54	37.15	8.152	27.4	0.695	5943.57	0	55.8	190.635	249.505	1.55	12404.5	25.665	0	2.495	559.03	5.32083	45
QH_S58	TS	2021	Qinghai-Tibet Plateau	96.54	37.12	7.082	14.77	0.338	2844.16	0	57.998	182.948	92.962	0.478	5485.72	0	0	0	229.864	5.32083	45

QH_S59	TS	2021	Qinghai-Tibet Plateau	96.54	37.12	7.087	21.8	0.394	4153.71	73.47	120.366	285.6	192.578	0.356	8454.19	0	0	0	235.884	5.32083	45
QH_S60	TS	2021	Qinghai-Tibet Plateau	96.54	37.12	9.028	0.324	0.0228	55.1973	0.6505	4.4015	1.3985	5.0578	1.1	50.2409	0.1314	0	0.0807	14.79	5.33333	45
QH_S61	TS	2021	Qinghai-Tibet Plateau	96.54	37.12	8.756	1.34	0.0524	267.455	3.8139	9.6658	4.3011	4.788	2.4363	277.654	0	0.3359	0.0591	64.6911	5.33333	45
QH_S62	TS	2021	Qinghai-Tibet Plateau	96.54	37.12	8.643	0.436	0.0331	69.7166	0.3848	5.6024	3.7639	7.3985	0.8336	65.3178	0.2939	0.2419	0.1142	20.0371	5.33333	45
QH_S63	TS	2021	Qinghai-Tibet Plateau	96.54	37.12	9.032	0.358	0.0346	56.2351	0.8319	6.2485	3.4751	6.9464	0.8566	47.9111	0.3495	0.2506	0.1517	13.5272	5.33333	45
QH_S76	DBXN	2021	Qinghai-Tibet Plateau	95.18	37.08	7.87	51.9	1.39	9800.51	0	34.38	1088.05	1840.39	1.095	25627.9	0	11.635	9.655	1102.88	4.77917	39
QH_S77	DBXN	2021	Qinghai-Tibet Plateau	95.18	37.08	7.904	35.5	1.38	6506.26	52.165	25.78	754.96	799.555	0	17534.1	28.295	11.4	8.58	155.61	4.77917	39
QH_S78	DBXN	2021	Qinghai-Tibet Plateau	95.18	37.08	7.727	52.1	1.935	9623	0	26.8	1288.83	1519.43	0.91	26758.7	0	11.97	10.475	278.665	4.77917	39
QH_S79	DBXN	2021	Qinghai-Tibet Plateau	95.18	37.08	7.645	94.7	2.895	20488.1	0	43.935	2152.34	2618.46	0	51930.8	0	12.59	17.625	441.185	4.77917	39
QH_S50	GH	2021	Qinghai-Tibet Plateau	97.36	37.07	8.74	1.435	0.0343	90.938	0.9955	9.5695	9.786	0	0.6804	137.502	0.2818	0.2383	0.0752	244.895	3.99167	50
QH_S51	GH	2021	Qinghai-Tibet Plateau	97.36	37.07	8.98	1.97	0.0566	307.139	2.9519	6.9953	24.7296	41.2406	0.5012	573.732	0	0.2656	0.1944	58.3875	3.99167	50
QH_S52	GH	2021	Qinghai-Tibet Plateau	97.36	37.07	8.793	2.35	0.0941	296.463	3.6894	7.772	18.4583	183.138	0.6835	426.783	0	0.3033	0.0608	265.864	3.99167	50
QH_S94	DDBX	2021	Qinghai-Tibet Plateau	95.1	37.03	9.321	0.758	0.0315	112.35	1.1666	3.4787	4.1944	25.6198	0.429	161.673	0.0416	0.2503	0.0656	50.78	3.99167	50
QH_S95	DDBX	2021	Qinghai-Tibet Plateau	95.1	37.03	8.647	2.51	0.1186	332.598	3.9385	13.0575	28.4508	162.892	0.7693	447.117	0.798	0.3083	0.0938	286.09	3.99167	50
QH_S96	DDBX	2021	Qinghai-Tibet Plateau	95.1	37.03	8.59	14.56	2.502	2708.09	1.03	125.228	220.532	29.418	8.694	5057.45	0	5.26	0.82	364.83	1.42083	76
QH_S100	SN	2021	Qinghai-Tibet Plateau	94.21	37	8.271	10.45	2.066	1968.02	1.15	126.664	136.362	19.916	6.006	3558.61	0	5.054	0.806	310.572	1.42083	76
QH_S101	SN	2021	Qinghai-Tibet Plateau	94.21	37	8.446	14.75	2.51	2682.27	0	118.47	263.304	40.574	6.632	5187.61	0	5.53	0	385.566	1.42083	76
QH_S98	SN	2021	Qinghai-Tibet Plateau	94.21	37	8.602	12.88	4.462	1801.63	12.344	177.652	492.592	196.004	4.318	4220.98	0	6.366	0.794	681.352	1.42083	76
QH_S99	SN	2021	Qinghai-Tibet Plateau	94.21	37	7.973	14.35	2.046	1507.43	1.592	71.64	772.06	319.408	0.774	5493.1	0	0	0.938	316.602	4.76667	39
QH_S134	CKI	2021	Qinghai-Tibet Plateau	98.07	36.59	8.068	15.66	2.31	1625.38	1.55	74.818	849.11	411.332	0.734	5962.21	0	0	0.808	409.718	4.76667	39
QH_S135	CKI	2021	Qinghai-Tibet Plateau	98.07	36.59	7.862	15.72	1.604	1790.77	14.336	77.554	663.686	677.384	0.72	5685.48	0	0	1.264	674.96	4.76667	39
QH_S136	CKI	2021	Qinghai-Tibet Plateau	98.07	36.59	7.331	14.65	3.416	1946.15	1.848	98.662	553.902	328.156	1.114	5274.09	0	0	0.93	514.498	5.17083	29
QH_S137	CKI	2021	Qinghai-Tibet Plateau	98.07	36.59	8.114	25.6	10.055	3598.98	0	270.13	1186.02	469.61	1.185	11641.5	3.07	0	2.335	987.07	5.17083	29
QH_S47	KK	2021	Qinghai-Tibet Plateau	98.17	36.58	8.76	1.621	0.0139	270.379	0	2.6922	1.5924	11.8762	1.43	209.385	0	0	2.02	159.085	15.5917	15
QH_S48	KK	2021	Qinghai-Tibet Plateau	98.17	36.58	8.24	30.3	0	6578.12	3.345	30.635	130.715	543.96	0.795	6896.89	0	0	0	3450.74	15.6333	14
QH_S43	XLG	2021	Qinghai-Tibet Plateau	98.27	36.49	8.37	46	0	9434.45	2.715	53.58	240.84	708.955	1.115	16228.9	0	11.395	0	3461.15	15.6333	14
QH_S45	XLG	2021	Qinghai-Tibet Plateau	98.27	36.49	8.25	88.4	0	20676.8	0	69.6	254.09	266.255	0	41343.8	0	11.56	1.635	6059.57	15.6333	14
QH_S46	XLG	2021	Qinghai-Tibet Plateau	98.27	36.49	8.32	12.87	0	2051.81	0.736	26.612	191.492	22.368	1.825	2518.13	0	11.395	2.545	1766.28	9.20417	153
QH_S64	SLGL	2021	Qinghai-Tibet Plateau	97.21	36.45	8.48	14.6	0	2127.63	1.38	38.202	191.978	18.416	1.515	2451.19	5.29	11.525	2.42	1162.36	9.20417	153
QH_S65	SLGL	2021	Qinghai-Tibet Plateau	97.21	36.45	8.38	13.75	0	2839.17	1.266	37.34	254.842	27.256	1.83	2926.28	8.73	11.58	2.55	1771.18	9.20417	153
QH_S66	SLGL	2021	Qinghai-Tibet Plateau	97.21	36.45	8.7	5.42	0	991.646	0	9.318	10.16	7.228	1.28	1044.64	0	0	0	468.085	7.02083	171
QH_S68	CEH1	2021	Qinghai-Tibet Plateau	95.11	36.45	9	9.13	0	1915.13	0	18.6207	33.5328	16.7622	1.53	1431.44	0	0	0	1041.38	7.02083	171
QH_S69	CEH1	2021	Qinghai-Tibet Plateau	95.11	36.45	8.43	0.714	0.0143	78.8076	0	3.7928	6.9773	32.7059	0.6512	98.1151	0	0.2531	0	48.0572	7.02083	171
QH_S70	CEH1	2021	Qinghai-Tibet Plateau	95.11	36.45	8.89	25	0.254	5574.47	43.878	71.608	363.13	47.254	2.18	4969.94	0	14.3	5.875	3988.71	3.54167	167
QH_S71	CEH1	2021	Qinghai-Tibet Plateau	95.11	36.45	8.92	17.67	0	3705.94	11.026	44.284	100.534	0	1.715	2538.47	2.28	12.485	2.435	2427.19	3.54167	167
QH_S130	CKA	2021	Qinghai-Tibet Plateau	99.11	36.41	8.56	2.36	0.0194	404.438	4.4694	10.104	3.9629	6.5975	2.045	394.105	2.53	0	2.545	168.4	3.54167	167
QH_S131	CKA	2021	Qinghai-Tibet Plateau	99.11	36.41	8.68	18.84	0	4257.29	43.14	56.818	212.044	0	1.27	3307.85	0	12.78	2.66	2755.38	3.54167	167
QH_S132	CKA	2021	Qinghai-Tibet Plateau	99.11	36.41	8.31	1.035	0.0158	158.026	0	5.6878	7.621	14.2889	1.9971	105.919	0	0.3731	0	104.417	5.8375	153
QH_S133	CKA	2021	Qinghai-Tibet Plateau	99.11	36.41	8.06	0.45	0.0136	72.846	0	2.8376	2.1086	4.623	1.42	43.6335	0	0.3288	0	28.4734	5.8375	153
QH_S122	QHH1	2021	Qinghai-Tibet Plateau	100.02	36.39	8.38	1.726	0.0199	300.545	0	10.9645	16.2824	20.5841	3.3726	220.28	0	0.6571	0	179.136	5.8375	153
QH_S123	QHH1	2021	Qinghai-Tibet Plateau	100.02	36.39	8.7	49	1.28	6834.25	46.695	43.07	475.46	195.51	4.55	3939	0	11.455	0	5519.71	9.35417	44
QH_S125	QHH1	2021	Qinghai-Tibet Plateau	100.02	36.39	7.38	2.67	0.0582	350.822	4.4877	8.3761	27.3018	113.723	1.465	271.085	0	0	0	301.025	9.35417	44
QH_S72	CEH2	2021	Qinghai-Tibet Plateau	95.03	36.39	7.71	14.11	0.262	1205.2	7.916	11.38	27.854	130.61	1.1	1689.64	0	0	0	431.225	9.35417	44
QH_S73	CEH2	2021	Qinghai-Tibet Plateau	95.03	36.39	7.99	6.29	0.55633	10438.6	0	59.587	283.809	595.219	0.84	7700.37	17.655	0	2.78	2103.59	9.3875	44
QH_S74	CEH2	2021	Qinghai-Tibet Plateau	95.03	36.39	8.17	4.74	0.268	1259.25	0.67	7.854	20.0093	240.374	0.835	6122.84	14.395	0	2.705	552.9	9.3875	44

QH_S75	CEH2	2021	Qinghai-Tibet Plateau	95.03	36.39	7.9	20.7	0.29	1318.75	0.678	12.0997	34.7487	182.296	0	6376.91	0	0	3.53	565.6	9.3875	44
QH_S126	QHH2	2021	Qinghai-Tibet Plateau	100.33	36.34	7.63	1.989	0	5.1427	0	0.2162	0.3811	1.2138	1.4	221.345	0	0	0	252.715	9.3875	44
QH_S127	QHH2	2021	Qinghai-Tibet Plateau	100.33	36.34	8.15	0.833	0.0142	129.355	0	2.4534	2.5938	12.5839	1.1384	142.381	0	0.2293	0	39.426	15.5917	15
QH_S128	QHH2	2021	Qinghai-Tibet Plateau	100.33	36.34	7.99	0.486	0.014	70.952	0	2.1677	2.1419	13.711	1.0633	33.2143	0	0	0	40.3943	15.5917	15
QH_S129	QHH2	2021	Qinghai-Tibet Plateau	100.33	36.34	7.9	4.5	0	726.14	0.76	8.604	15.446	89.452	1.33	900.98	3.295	0	1.965	368.32	15.5917	15

Supplementary Table 2. Mantel tests of environmental factors against the β-NTI of abundant, intermediate, and rare taxa in alkaline lake sediments. Asterisks indicate the level of statistical significance: * $P < 0.05$, ** $P < 0.01$, *** $P < 0.001$.

Environmental factors	Abundant		Intermediate		Rare	
	r	significance	r	significance	r	significance
pH	0.0984	***	0.2712	***	0.1652	***
EC	0.0902	**	0.1300	***	0.1125	***
Li-	-0.0430		0.1364	**	0.1700	***
Na+	0.1207	***	0.1606	***	0.1528	***
NH4+	-0.0487		0.0322		0.0646	*
K+	-0.0097		0.1610	***	0.1540	***
Mg2+	0.0014		0.1227	**	0.0963	**
Ca2+	0.0430		0.1201	**	0.1290	***
F-	0.0603		0.1455	***	0.1035	**
Cl-	0.0741	*	0.1344	***	0.1270	***
NO2-	0.0392		0.0257		-0.0383	
Br-	0.1006	**	0.1130	**	0.0327	
NO3-	0.0691	*	0.0657		-0.0116	
MAT	0.2112	***	0.2269	***	0.0053	
MAP	0.0240		0.2185	***	0.0020	
Spatial	0.2777	***	0.3190	***	0.0569	

Supplementary Table 3. Partial mantel test of the relationships between phylogenetic turnover (β-NTI) and potential environmental predictors on rare taxa after controlling for other environmental factors in sediments of alkaline lakes. Asterisks indicate the level of statistical significance: ∗ $P < 0.05$, ∗∗ $P < 0.01$, ∗∗∗ $P < 0.001$.

Effect of selected variables on β-NTI	Controlling for	Mantel statistic r	significance
pH	Spacial + Environmental factors (excl. pH)	0.1629	***
spatial	Spacial + Environmental factors (excl. pH) + pH	0.0579	
Environmental factors (excl. pH)	Spacial + pH	0.0230	
Li+	Spacial + Environmental factors (excl. Li+)	0.0007	
K+	Spacial + Environmental factors (excl. K+)	0.0962	
Na+	Spacial + Environmental factors (excl. Na+)	0.0667	
EC	Spacial + Environmental factors (excl. Li+)	0.0256	
F-	Spacial + Environmental factors (excl. F-)	0.1175	**
Ca2+	Spacial + Environmental factors (excl. Ca2+)	0.0769	
Cl-	Spacial + Environmental factors (excl. Cl-)	0.0190	

Supplementary Table 4. Partial mantel test of the relationships between phylogenetic turnover (β-NTI) and potential environmental predictors on abundant taxa after controlling for other environmental factors in sediments of alkaline lakes. Asterisks indicate the level of statistical significance: ∗ $P < 0.05$, ∗∗ $P < 0.01$, ∗∗∗ $P < 0.001$.

Effect of selected variables on β-NTI	Controlling for	Mantel statistic r	significance
Spatial	Environmental factors (excl. spatial factors)	0.2099	***
MAT	Spatial + Environmental factors (excl. MAT)	0.1755	***
Environmental factor (excl. MAT)	Spatial+MAT	0.0934	*
pH	Spatial + Environmental factors (excl. pH)	0.0518	
Na+	Spatial + Environmental factors (excl. Na+)	0.0843	

Supplementary Table 5. Relative abundance and percentage of total genes based on predictions using PICRUSt2 and Tax4Fun, repectively.

Pathway	Description	Abundant taxa		Rare taxa	
		PICRUSt2	Tax4Fun	PICRUSt2	Tax4Fun
ko00710	Carbon fixation in photosynthetic organisms	8313.486	0.296%	3208.061	0.255%
ko00720	Carbon fixation pathways in prokaryotes	8350.150	0.760%	3204.333	0.764%

Supplementary Table 6. Partial mantel test of the relationships between phylogenetic turnover (β-NTI) and potential environmental predictors on intermediate taxa after controlling for other environmental factors in sediments of alkaline lakes. Asterisks indicate the level of statistical significance: * $P < 0.05$, ** $P < 0.01$, *** $P < 0.001$.

Effect of selected variables on βNTI	Controlling for	Mantel statistic r	significance
pH	Environmental factor (excluding pH)+spatial	0.2456	***
spatial	Environmental factor (excluding pH)+pH	0.2937	***
Environmental factor (excluding pH)	pH+spatial	0.4486	
K^+	Environmental factor (excluding K^+)+*spatial*	0.0832	
Na^+	Environmental factor (excluding Na^+)+*spatial*	-0.0491	
EC	Environmental factor (excluding Li^+)+*spatial*	-0.1065	
F^-	Environmental factor (excluding F^-)+*spatial*	0.1164	**
Cl^-	Environmental factor (excluding Cl^-)+*spatial*	-0.0878	
MAT	Environmental factor (excluding MAT)+spatial	0.1258	***
Environmental factor (excluding MAT)	Spatial+MAT	0.1495	***
MAP	Environmental factor (excluding MAP)+spatia	0.09215	**
